# Dispersion of Activation in Single‐Beat Global Maps During Programmed Ventricular Stimulation Identifies Infarct‐Related Ventricular Tachycardia Isthmus Sites

**DOI:** 10.1161/JAHA.124.038441

**Published:** 2024-11-22

**Authors:** Andrés Redondo‐Rodríguez, Alba Ramos‐Prada, Jorge G. Quintanilla, David Calvo, Javier Sánchez‐González, Daniel Enríquez‐Vázquez, Manuel Marina‐Breysse, Jose Manuel Alfonso‐Almazán, Juan José González‐Ferrer, Victoria Cañadas‐Godoy, Ricardo Salgado‐Aranda, Carlos A. Morillo, Julián Pérez‐Villacastín, Nicasio Pérez‐Castellano, David Filgueiras‐Rama

**Affiliations:** ^1^ Centro Nacional de Investigaciones Cardiovasculares (CNIC) Novel Arrhythmogenic Mechanisms Program Madrid Spain; ^2^ Fundación Interhospitalaria para la Investigación Cardiovascular Madrid Spain; ^3^ Centro de Investigación Biomédica en Red de Enfermedades Cardiovasculares (CIBERCV) Madrid Spain; ^4^ Instituto de Investigación Sanitaria del Hospital Clínico San Carlos (IdISSC) Cardiovascular Institute Madrid Spain; ^5^ Philips healthcare Iberia Madrid Spain; ^6^ Complexo Hospitalario Universitario A Coruña. Servicio de Cardiología, Instituto de Investigación Biomédica A Coruña (INIBIC) A Coruña Spain; ^7^ Department of Cardiac Sciences, Libin Cardiovascular Institute, Cumming School of Medicine University of Calgary Calgary Alberta Canada

**Keywords:** dispersion of activation, electroanatomical mapping, magnetic resonance imaging, programmed ventricular stimulation, single‐beat mapping, ventricular arrhythmia, ventricular tachycardia isthmus, Electrophysiology, Animal Models of Human Disease, Myocardial Infarction

## Abstract

**Background:**

Electrophysiological characterization of ventricular tachycardia (VT) isthmus sites is complex and time‐consuming. We aimed at developing and validating a global mapping strategy during programmed ventricular stimulation (PVS) to reveal the underlying electrophysiological properties of the infarct‐related substrate and to enable identification of highly heterogeneous activation sites associated with protected VT isthmus sites.

**Methods and Results:**

Experimental study that included 22 pigs with established myocardial infarction undergoing in vivo characterization of the anatomical and functional myocardial substrate associated with potential arrhythmogenicity. High‐density sequential activation maps during ventricular pacing and VT were compared with single‐beat maps using a 64‐pole basket catheter positioned in the left ventricle. Further analyses were performed using a novel local activation time‐dispersion score to identify regional activation time heterogeneities on both baseline drive pacing and each of the extrastimuli of the PVS protocol. Basket catheter splines covered a median of 81.2% of the endocardial surface of the left ventricle. Basket‐catheter‐derived single‐beat activation maps (N=16) during pacing showed a linear relationship with high‐density sequential activation maps. Induction of ventricular arrhythmias was associated with higher local activation time‐dispersion score values on single‐beat global maps during PVS (N=6, 46 arrhythmia induction attempts). Single‐beat‐derived local activation time‐dispersion score maps during successive coupled extrastimuli of the PVS showed a progressive increase in the predictive performance to identify monomorphic VT isthmus sites within the scar region (area under the curve = 0.779 in S2, area under the curve = 0.859 in S4; N=7).

**Conclusions:**

Sixty‐four‐pole‐derived single‐beat local activation time‐dispersion score global maps during PVS identify infarct‐related endocardial regions with highly heterogeneous activation times that are associated with protected VT isthmus sites.

**Nonstandard Abbreviations and Acronyms**
CL: cycle length
LAT: local activation time
LAT‐DS: local activation time ‐ dispersion score
PVS: programmed ventricular stimulation

**Clinical Perspective**

**What Is New?**

- Single‐beat global endocardial mapping from large multipolar basket catheters provides rapid and reliable information of endocardial left ventricular activation patterns during programmed ventricular stimulation.
- A novel local activation time dispersion score, applied during programmed ventricular stimulation, identifies infarct‐related endocardial regions with highly heterogeneous activation times that are associated with protected ventricular tachycardia isthmus sites in pigs.
- Average local activation time dispersion score values on single‐beat maps during programmed ventricular stimulation show a positive correlation with poststimulation arrhythmia severity.

**What Are the Clinical Implications?**

- Local activation time dispersion score analysis of single‐beat maps during programmed ventricular stimulation may help to efficiently identify endocardial regions associated with protected isthmus sites in clinical scenarios with poorly tolerated or unmappable ventricular tachycardia episodes.

Reentry is the most common mechanism associated with ventricular tachycardia (VT) maintenance in patients with infarct‐related myocardial substrate.^1^ Reentrant VT circuits can be represented in a simplified manner to describe the main structural components and electrophysiological properties necessary for self‐perpetuation of wavefront propagation throughout a closed rotational circuit.^2^ Indeed, a critical feature of reentrant VT circuits is a wavefront propagation through protected channels of conducting tissue (ie, isthmus site) isolated from the rest of surrounding myocardium during VT.^3^ Identification of such protected isthmus sites is the main target for effective VT termination.^2^ In clinical practice, complete characterization of reentrant VT circuits and identification of critical isthmus sites using classical electrophysiological criteria and high‐density mapping during tachycardia is complex.^4^ VTs are often unmappable, and such isthmus sites cannot be identified because of hemodynamic instability. These limitations have motivated the implementation of mapping strategies during sinus rhythm or ventricular pacing aiming to identify potentially arrhythmogenic scar regions that may be associated with VT isthmuses.^5^, ^6^, ^7^ Nonetheless, sequential mapping strategies require expert operators and time‐consuming procedures.

Importantly, VT initiation requires the presence of unidirectional conduction block with successful wavefront propagation in only 1 direction to complete the rotational circuit.^8^ The initial unidirectional block may occur in areas with anatomical obstacles within the infarcted myocardium, but it can also be functional in highly heterogeneous myocardial regions.^9^, ^10^ Interestingly, the presence of decremental conduction during ventricular pacing with extrastimuli has been shown to precede unidirectional block in myocardial regions critical for VT onset and maintenance.^11^, ^12^ Such decremental potentials can be detected using near field single‐beat global mapping on the endocardial surface and provide high specificity (95%) although low sensitivity (29%) for detecting VT isthmus sites.^11^ Therefore, there is a need to improve sensitivity of near field single‐beat approaches for detecting VT isthmus sites and, eventually, make them useful in the clinic.

We hypothesize that myocardial regions with large activation time dispersion on single‐beat multipolar recordings during programmed ventricular stimulation (PVS) are sensitive and specific for detecting protected isthmus sites of infarct‐related monomorphic VT episodes. Moreover, the severity of poststimulation outcomes classified as noninducible arrhythmia, monomorphic VT, polymorphic VT, or ventricular fibrillation (VF) will be associated with a progressively higher degree of activation time dispersion. To test these hypotheses, we used a translational experimental porcine model of myocardial infarction, quantified local activation times and their local dispersion and finally derived a local activation time dispersion score (LAT‐DS) on single‐beat endocardial global maps during PVS.

## METHODS

The data that support the findings of this study are available from the corresponding author upon reasonable request. Full details of the novel analytical methods used in this article are fully disclosed in the article and in Data S1.

### Experimental Protocol

The study included 22 pigs (strain Large‐white, males/females 18/4) with established myocardial infarction in the anterior wall. The pigs (median age 3 months) underwent ischemia–reperfusion in the left anterior descending coronary artery, as reported elsewhere.^13^ A median of 9 weeks (interquartile range, 8–12 weeks) later, all pigs (weight 60.2 kg [interquartile range, 52.6–73.0 kg]) underwent left ventricular substrate characterization using 3‐dimensional late‐gadolinium enhancement cardiac magnetic resonance (LGE‐CMR) imaging. Then, the animals underwent an invasive procedure for electrophysiological characterization of the arrhythmogenic substrate and VT mapping. All procedures were performed with the pigs under general anesthesia (intravenous ketamine 2 mg/kg per h, xylazine 0.2 mg/kg per h, and midazolam iv 0.2 mg/kg per h) and were approved by the competent ethical authority (Comunidad de Madrid, Ref#PROEX097/17). This was a pilot experimental study and the sample size was estimated to ensure the use of the minimum number of animals. No a priori exclusion criteria were used, and no animals were excluded after experimental completion. Animals were allocated to 1 of 2 experimental groups in a nonrandomized sequential manner. No intergroup comparisons were performed and no possible confounding factors were identified. Animal experiments complied with ARRIVE (Animal Research: Reporting of In Vivo Experiments) guidelines,^14^ and were performed at Centro Nacional de Investigaciones Cardiovasculares (Madrid, Spain).

### Magnetic Resonance Imaging Acquisition and Processing

LGE‐CMR imaging acquisition was performed with an Achieva 3T‐Tx wholebody scanner (Philips Healthcare, Best, The Netherlands) equipped with a 32‐element and phased‐array cardiac coil. Seven minutes after intravenous contrast injection of 0.2 mmol/kg gadoteric acid (Dotarem, Guerbet, France), 3‐dimensional LGE‐CMR sequences were acquired using an inversion‐recovery spoiled turbo field echo with isotropic resolution of 1.5×1.5×1.5 mm. LGE‐CMR images were then processed using the ADAS3D software (v.2.11.0, Adas3D Medical S.L., Barcelona, Spain) for scar identification based on the full‐width‐half‐maximum method, which normalizes signal intensity to maximum myocardial signal intensity.^15^ More specifically, signal intensity cut‐off values of 0.45 and 0.67 were used for detecting heterogeneous and dense scar, respectively.^16^

### Invasive Electrophysiological Study

**Figure 1:**
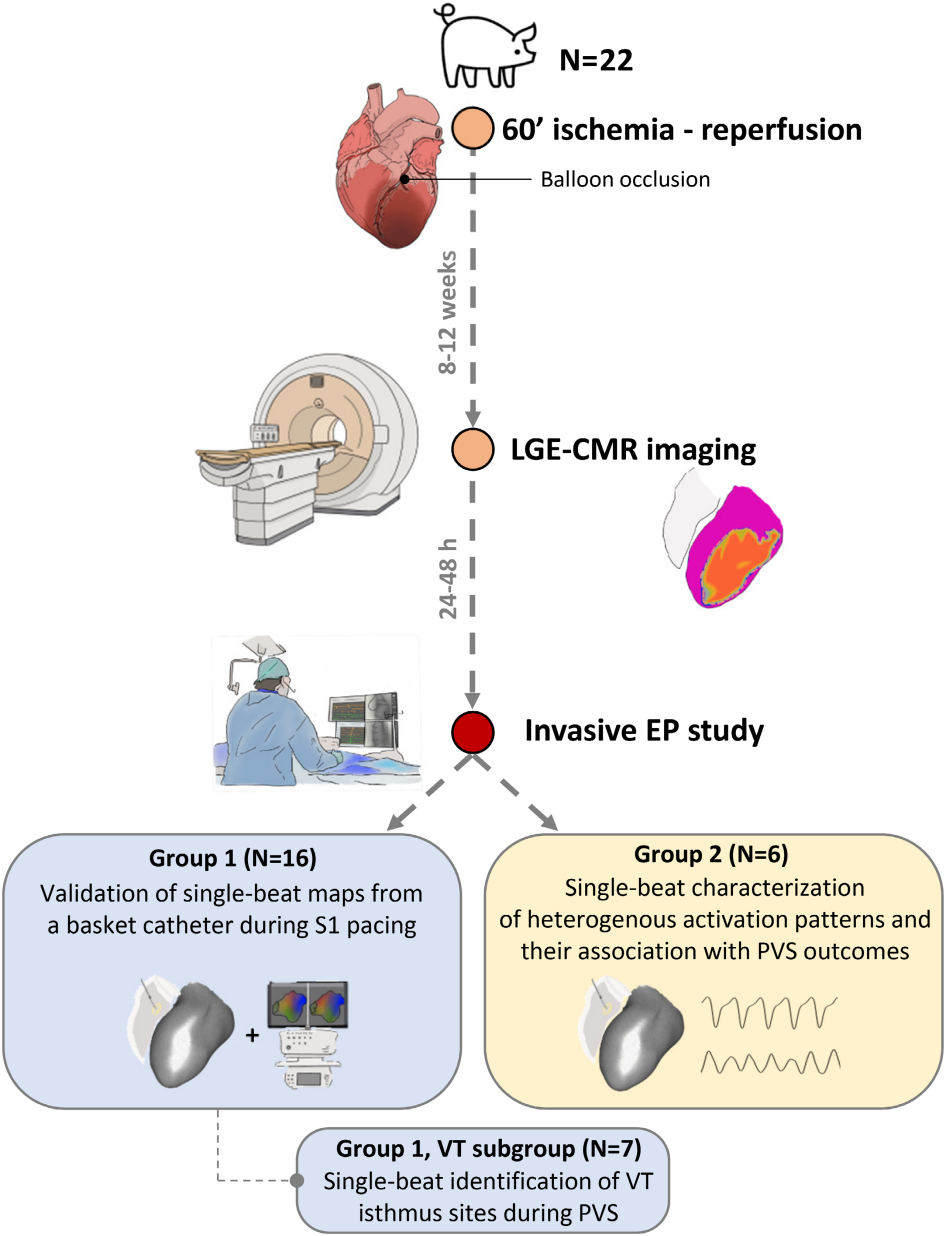
Schematic workflow of the experimental study. EP study indicates electrophysiological study; LGE‐CMR, late‐gadolinium enhancement cardiac magnetic resonance; PVS, programmed ventricular stimulation; and VT, ventricular tachycardia.

The animals underwent percutaneous venous and arterial femoral access to reach the right and left ventricles, respectively. In 16 pigs (Group 1, Figure 1), 3D electroanatomical guidance was used (Carto3 system, Biosense Webster, Diamond Bar, CA) to map the right and left ventricles during basic drive ventricular pacing (at 500 milliseconds cycle length [CL]) and generate high‐density sequential activation maps with a 3.5‐mm irrigated‐tip mapping/ablation catheter (Navistar Thermocool, Biosense Webster). Stability of the pacing site throughout the electrophysiological study was ensured by a screw‐in catheter in the right ventricle. Afterwards, a 64‐pole basket catheter (8‐spline 8‐electrode/spline, 5‐mm interelectrode spacing, Constellation, Boston Scientific, Lowell, MA) was positioned into the left ventricle to obtain single‐beat activation maps from the same pacing site in the right ventricle. The basket‐catheter was introduced through a long‐steerable sheath (8.5 F Agilis NxT Steerable Introducer, 71 cm lumen length, Abbott, St. Paul, MN) using the arterial access in the femoral artery. Two additional 24‐pole catheters (Orbiter ST, Boston Scientific) were positioned in the right ventricle towards the septum and the outflow tract. The latter aimed to record electrograms from potentially infarcted regions on the right septal side and from regions close to the pacing site. After the basic drive pacing protocol, PVS was performed to induce ventricular arrhythmia using a basic drive CL (S1; 10 pacing beats) at 350, 300, 280, 260, and 250 milliseconds and a maximum of 4 coupled extrastimuli (S2, S3, S4, S5). At each basic drive CL, the extrastimuli were progressively decremented in 10 milliseconds steps until VT was induced, refractoriness or a 180 ms coupling interval. Electrograms from the basket catheter during PVS, VT induction, and VT were recorded with the Labsystem PRO EP recording system (Boston Scientific). Invasive blood pressure and oxygen saturation were continuously monitored during the procedure to assess hemodynamic tolerance during VT episodes.

In an additional group of animals (Group 2, N=6, Figure 1), the electrophysiological study included PVS with a minimum of 5 inducibility attempts to assess heterogeneity on single‐beat activation patterns and their association with poststimulation outcomes, which were classified as noninducible arrhythmia, sustained monomorphic VT, or polymorphic VT/VF. Further details are included in Data S1.

### Identification and Delineation of VT Isthmus Sites

In animals from Group 1, if the induced VT episode after PVS was well tolerated, additional sequential high‐density activation maps were obtained with the mapping/ablation catheter or a 20‐pole mapping catheter (PentaRay, Biosense Webster) to identify VT isthmus sites (VT subgroup, Figure 1). Activation maps were processed to identify and delineate VT regions of interest that included the protected isthmus site. The reference for assigning activation times during VT was the QRS onset. The VT region of interest was defined as the area with activation times from the latest activation point to −35% of the tachycardia CL and from the earliest activation point to +10% of the tachycardia CL, including also the junction between the earliest and latest activation points. If possible, entrainment maneuvers were performed to confirm the location of VT isthmus sites.

### Basket Catheter Location on the 3D Ventricular Space

Direct visualization of basket catheter electrodes on the 3D electroanatomical mesh generated with the Carto3 system was not possible. Therefore, orthogonal fluoroscopic images (antero‐posterior and left‐lateral views) of the catheters were processed off‐line with custom‐made software in Matlab (MathWorks Inc., Natick, MA). The software enabled the operator to manually annotate the electrode positions on each of the fluoroscopic views captured during the electrophysiology study. Electrode positions were projected onto the endocardial geometry of the left ventricular electroanatomical mesh after registration with the fluoroscopic images (Figure S1). The left ventricular geometry from LGE‐CMR images was also registered onto the electroanatomical geometry to project and visualize the infarct‐related scarring. Further details are provided in Data S1 and Figure S1.

### Annotation of Local Activation Times

**Figure 2:**
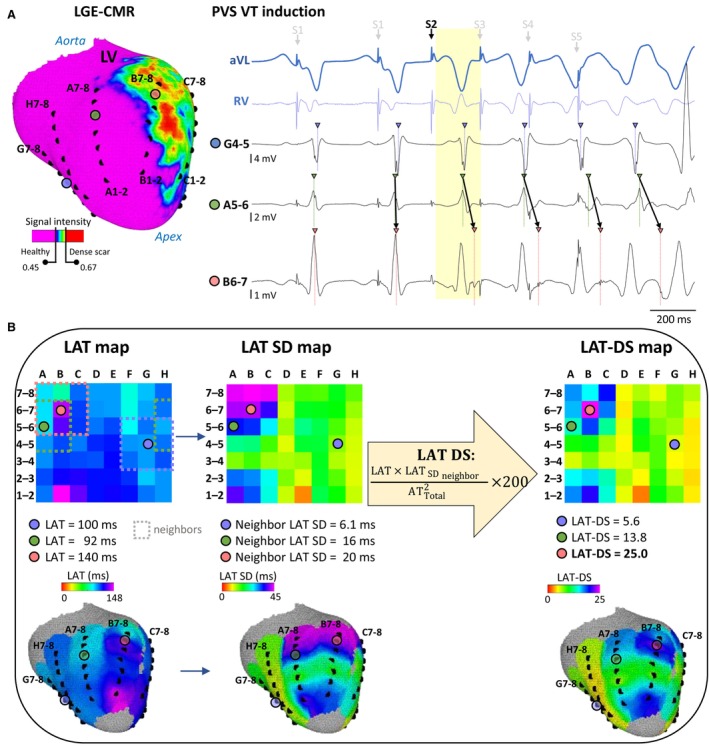
Local activation time‐dispersion score analysis for identification of potentially arrhythmogenic regions. **A**, Left, sample visualization of the basket‐catheter electrodes on the left ventricular (LV) endocardial surface. Electrodes are labeled with a letter to define the spline (A‐to‐H) and two numbers to define intra‐spline bipole from distal (1‐2) to proximal (7‐8). The infarct‐related substrate is derived from late‐gadolinium‐enhancement cardiac magnetic resonance (LGE‐CMR) images. Right, surface ECG, and representative electrograms from an electrode in the right ventricle (RV) close to the stimulation site and basket‐catheter electrodes (color‐coded circles in blue, green and light red) from 3 positions of the LV endocardial surface during programmed ventricular stimulation (PVS). Late potentials in the basket‐catheter bipole B6‐7 show a progressive delay during the PVS protocol that eventually leads to monomorphic ventricular tachycardia. Electrodes are highlighted with colored circles. (**B**) Local activation time (LAT), LAT‐SD (standard deviation), and local activation time dispersion score (LAT‐DS) maps of the S2 beat are represented from left to right, respectively, in 2D (top row) and 3D (bottom row). LAT values are calculated for all electrodes in the basket catheter. The LAT‐DS was calculated as the product of its LAT and the neighboring SD of LATs, normalized to the squared total single‐beat activation time. The total activation time is calculated considering the earliest right ventricle LAT value. Neighboring electrograms and its annotations for bipoles B6‐7 and G4‐5 are shown in Figure S2. The analysis identifies the electrode locations with the highest LAT‐DS values (eg, light red circle), which are the regions potentially sensitive for VT isthmus sites. AT_Total_ indicates total activation time; aVL, augmented vector left ECG lead; and MV, mitral valve.

Local activation times (LAT) from each of the poles of the basket catheter were annotated during PVS and VT, and referenced to the maximum deflection peak (positive or negative) of the QRS complex in one of the surface ECG leads. Bipolar LAT were annotated using maximum absolute peak criteria,^17^ and manually adjusted for late low‐voltage fractionated potentials inside or near infarct‐related scar tissue, as these more likely display local activations rather than large deflections indicative of far‐field ventricular activity (Figure 2A). Electrodes displaying poor contact with overt far‐field signal were discarded.^17^, ^18^ LATs on single‐beat maps were compared with higher resolution sequential LAT maps for validation of electrode positioning from single‐beat maps on the 3D left ventricular endocardial surface. LAT values from basket‐catheter bipoles were reviewed by an operator blinded to the LAT values from high‐resolution sequential maps and blinded to which bipoles were located inside the VT region of interest. Further details are provided in Data S1.

### Calculation of Local Activation Time‐Dispersion Score

In an effort to identify electrodes located within slow‐conduction regions with potential arrhythmogenicity, we defined a parameter named LAT‐DS. We propose that such regions may be especially characterized by large activation time dispersion (Figure 2A and 2B), which increases the risk of unidirectional block and reentry. Therefore, for each electrode of the basket catheter, LAT‐DS was calculated as the product of its LAT and the standard deviation of neighboring LATs, and normalized to the square of the total single‐beat activation time. The resulting value yielded a unitless score, which was then multiplied by a factor of 200 to draw a final score ranging from 0 to 100. Neighboring electrodes were defined as those at immediate adjacent poles and splines. A schematic 2D representation of a basket catheter and the neighboring definition are shown in Figure 2B. The complete process for LAT‐DS calculation of a given beat is also shown in Figure 2B and Figure S2. A spatially weighted alternative of the LAT‐DS (ie, LAT‐DSsw) accounting for the potentially variable distance between neighboring electrodes was also explored. In the latter, LAT values from more distant neighboring electrodes had less influence on the standard deviation of all neighboring LATs for a given electrode of the basket catheter. Further details are provided in Data S1 and in Figure S3.

### Statistical Analysis

Data are expressed as median and interquartile range for quantitative variables. Data normality was assessed with the Shapiro–Wilk test. Statistical significance was assessed by the *t* test or the Mann–Whitney/Wilcoxon test, as appropriate. ANOVA and Tukey's correction were used for post hoc comparisons between multiple groups. The Passing‐Bablok regression method was used to validate the linear relationship between sequential and single‐beat LAT values during S1 pacing. The area under the curve of receiver operating characteristic curves was used to assess the predictive performance of LAT and LAT‐DS during PVS to discern scarring regions outside the VT circuit from myocardial substrate inside the VT region of interest. Area under the curve values are expressed with their 95% CIs. Comparisons between receiver operating characteristic curves were tested using the DeLong's test. A *P* <0.05 was considered statistically significant for group comparisons. Analyses were performed with GraphPad Prism9 (GraphPad Software Inc., CA) or Matlab.

## RESULTS

### Myocardial Substrate in Infarcted Animals

LGE‐CMR imaging showed that infarct‐related scarring was mainly located in the septal and anterior wall regions of the left ventricle, affecting 23.2% (16.0, 31.6%) of the innermost layer of the left ventricular wall (ie, 10% of the myocardial wall thickness). A median of 14 electrodes (9.75, 20.5 electrodes) from the multipolar basket‐catheter were identified within infarct‐related scar regions. Electrograms from the 24‐pole catheters placed in the right ventricle did not include areas with infarct‐related substrate. Therefore, electrograms from the 24‐pole catheters were discarded for the analysis of local activation time dispersion.

### Left Ventricular Single‐Beat Activation Maps Highly Correlate With Sequential Activation Maps

Sixteen animals from Group 1 underwent sequential mapping (316 LAT points [153, 874 LAT points]) of the left ventricular LAT during right ventricular pacing. Additional single‐beat activation maps were obtained with the 64 electrodes of the basket catheter positioned in the left ventricle. Figure S4A shows a sample activation map derived from the 64‐electrode basket catheter and its corresponding higher‐resolution sequential activation map during ventricular pacing at 500 milliseconds cycle length. In such sample case, positioning of basket‐catheter electrodes and their mean absolute and relative errors are also shown in Figure S4B. Overall, single‐beat maps from the basket catheter covered a median of 77.1% (69.7, 87.0%) of the total left ventricular activation time documented in higher‐density sequential activation maps (Figure S4C). Single‐beat maps showed a mean absolute error of 5.1 milliseconds (3.9, 5.7 milliseconds) and a relative error of 6.4% (4.8%, 7.4%) compared with LAT values at the same location from higher‐resolution sequential activation maps (Figure S4D). Passing‐Bablok analysis confirmed a linear relationship between LAT values on single‐beat maps and their corresponding values on higher‐resolution sequential maps (*y*=1.020*x* + 0.876, Figure S4E).

The splines of the basket catheter were able to cover a median of 81.2% (68.3, 82.7%) of the endocardial surface of the left ventricle with a 5‐mm interelectrode spacing within individual splines and a 22.1 mm (20.7, 22.9 mm) interspline distance (Figure S5A and S5B). Overall, these results indicate that single‐beat activation maps from large multipolar basket catheters provide rapid and reliable information of endocardial left ventricular activation patterns during pacing.

### VT Isthmus Sites Are Located in Regions With Higher Than Surrounding Local Activation Time Dispersion Score Values

**Figure 3:**
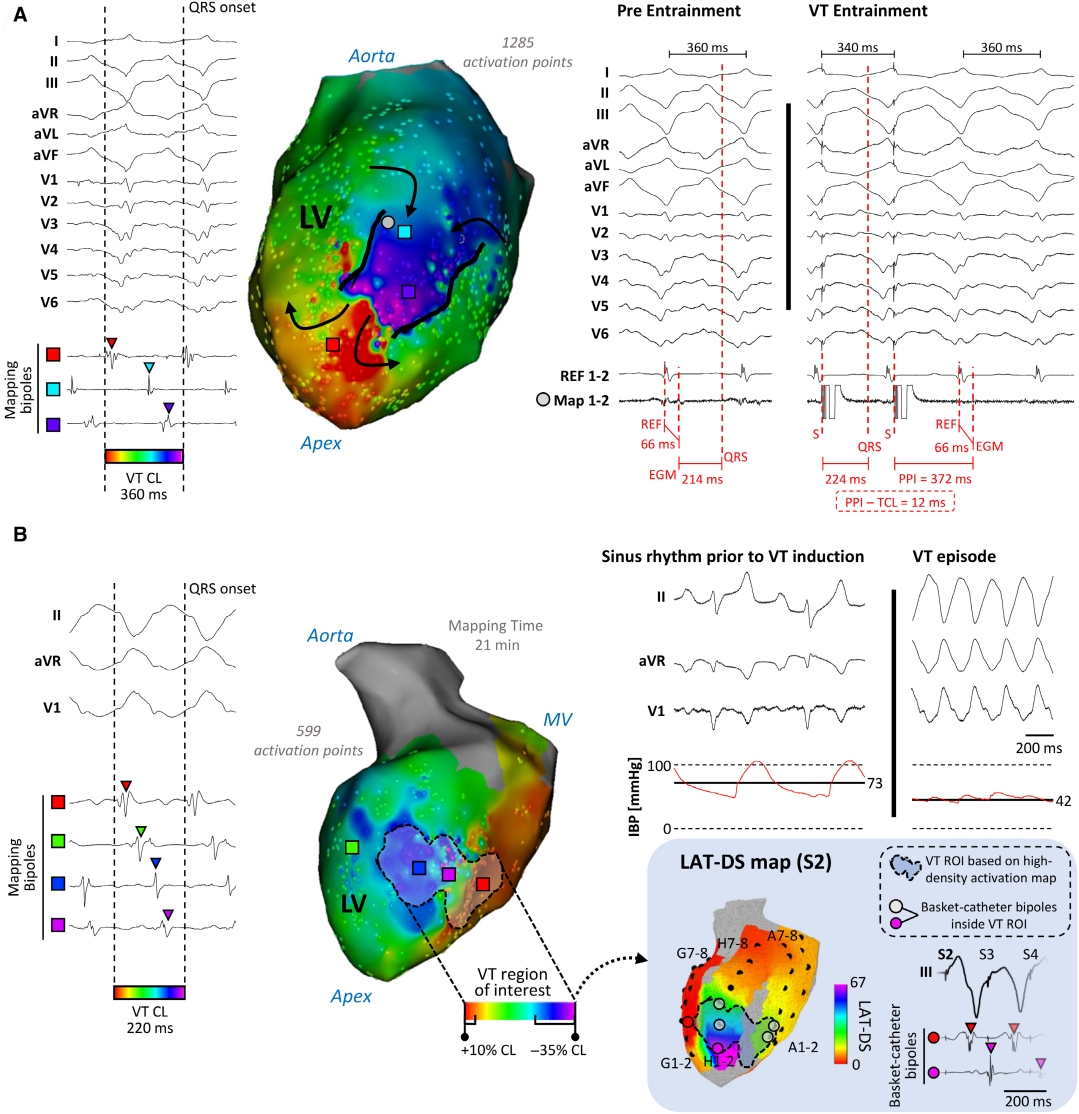
Electrophysiological identification of ventricular tachycardia regions of interest. **A**, Sample electrophysiological characterization of a reentrant ventricular tachycardia (VT) circuit. Left, 12‐lead ECG during VT and sample electrograms at different mapping locations on the color‐coded high‐density activation map. Black arrows indicate the figure‐of‐eight reentrant circuit. Right, entrainment was performed from the gray circle on the activation map. A screw‐in catheter in the right ventricle (REF) was used as a stable reference to identify timing of the low amplitude electrogram on the mapping catheter. Both activation mapping and entrainment confirmed an isthmus site location. (**B)** Sample electrophysiological characterization of a VT circuit with a shorter cycle length (CL) (220 milliseconds). Left, 3 surface ECG leads during VT and sample electrograms at different mapping locations on the color‐coded high‐density activation map. On the map, the VT region of interest (ROI), containing the isthmus site is represented with a black dashed line. Top right, invasive blood pressure (IBP) values on the femoral artery during sinus rhythm and VT. Bottom right, local activation time dispersion score (LAT‐DS) map of the first coupled extrastimulus (S2) of the programmed ventricular stimulation before VT induction. Basket‐catheter bipoles inside the VT ROI are highlighted with gray and purple circles. Sample electrograms from basket catheter bipoles inside (in purple) and outside (in red) the VT ROI are also shown. The mapping time in (**B**) was 21 minutes, although the VT terminated spontaneously in 2 occasions during mapping. The same VT morphology was consistently reinduced upon programmed ventricular stimulation, which enabled the characterization of the VT isthmus site. aVF, aVL and aVR indicate augmented vector foot, left and right ECG leads, respectively; EGM, electrogram; LV, left ventricle; MV, mitral valve; PPI, post‐pacing interval; and TCL, tachycardia cycle length.

Sequential activation maps (218 valid LAT points [164, 520 LAT valid points]) enabled to fully characterize 7 VT morphologies in 7 animals from Group 1 (VT subgroup). The mapping time during VT was 11.0 minutes (8.9, 15.5 minutes) with mean invasive blood pressure values of 52.8 mm Hg (39.7, 62.9) during mapping. Activation maps included electrograms covering 88.1% (86.0, 92.6%) of the VT CL. Further entrainment maneuvers at protected isthmus sites were also possible in 3 animals. Sample maps and tracings with the electrophysiological criteria to identify VT isthmus sites are shown in Figure 3A and 3B. Complete information on the characterization of VT episodes from all cases is shown in Table 1. Single‐beat maps obtained with the basket catheter during VT were sufficient to map 78.8% (59.7, 84.2%) of VT CL (Figure S5C and S5D).

**Table .** Electrophysiological Characterization of VT Circuits

Animals from Group 1, VT subgroup (N=7)	
VT cycle length, ms	228 (205–256)
VT circuit characterized with activation mapping, n (%)	7 (100)
Valid mapping points, n	218 (164–520)
Mapping time, min	11.0 (8.0–15.5)
Percentage of the VT cycle length mapped	88.1 (86.0–92.6)
Average IBP during VT mapping, mm Hg	52.8 (39.7–62.9)
VT circuit characterized with entrainment, n (%)	3 (42.8)

Data are expressed as median and interquartile ranges and n (%), as appropriate. IBP indicates invasive blood pressure; and VT ventricular tachycardia.

In 6 of 7 animals, at least 1 of the 64‐basket‐catheter electrodes (median 3 electrodes [2.25, 5.75 electrodes]) was identified within the VT region of interest containing the isthmus site on sequential higher‐resolution activation maps. A sample case of basket‐catheter electrodes located inside the identified region of interest is illustrated in Figure 3B. In fact, these electrodes showed higher than surrounding LAT‐DS values during the S2 of the PVS before VT induction (Figure 3B). In 1 case the basket catheter did not include any electrode inside the identified VT isthmus site because the circuit was located in the left ventricular apex, within a region without electrodes on the catheter tip.

**Figure 4:**
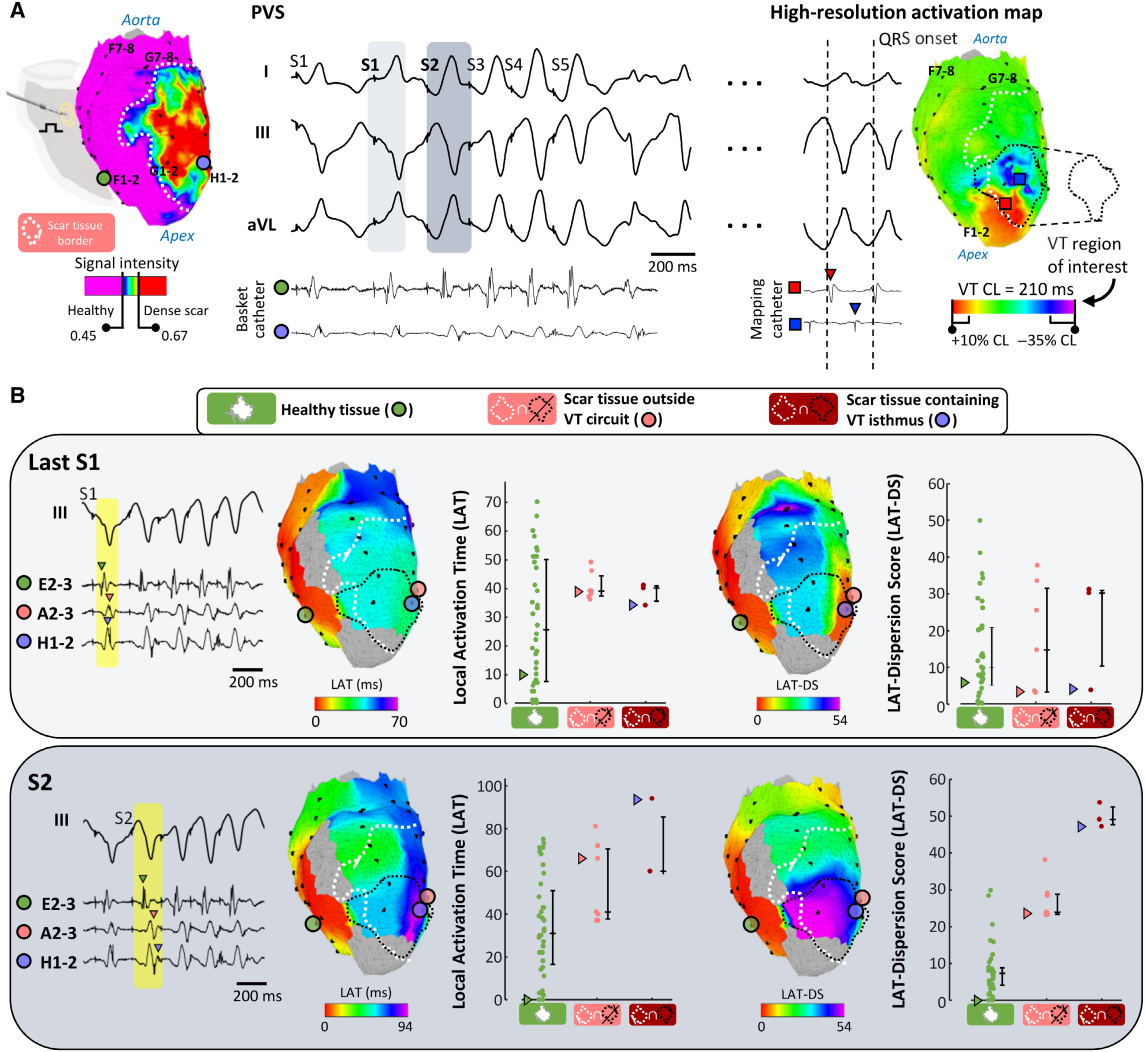
Local activation time‐dispersion score analysis on single‐beat maps identifies ventricular tachycardia isthmus sites. **A**, Left, sample case showing a left ventricular scar‐characterization based on late gadolinium enhancement cardiac magnetic resonance imaging and a schematic view of the pacing site in the right ventricle. Representative basket‐catheter bipoles from inside and outside the scar region are represented with blue and green colored circles, respectively. Middle, programmed ventricular stimulation (PVS) with 4 coupled extrastimuli (S2, S3, S4, S5) yielding monomorphic ventricular tachycardia (VT) induction. Electrograms from the representative bipoles are shown below surface ECG. Right, VT circuit characterization based on sequential activation mapping. The reference for assigning activation times during VT was the QRS onset. The scar contour is represented with a white dashed line. The VT region of interest (containing the isthmus site) is defined using the +10% to −35% of VT cycle length (VT CL) and is represented with a black dashed line. Colored squares indicate sample activation points and electrograms from inside the VT region of interest. Black dots indicate basket‐catheter electrodes on each of the visible splines on the right anterior oblique view (55°). (**B)** Sample analysis of local activation times (LAT) (left) and LAT‐dispersion score (LAT‐DS) values (right) of the last S1 of the basic drive pacing at 350 milliseconds CL (upper row) and the first coupled extrastimulus (S2, at 240 milliseconds coupling, bottom row). Basket‐catheter electrodes are divided into 3 regions: inside healthy myocardium (n=45), inside a scarring region outside the VT region of interest (n=8) and inside a scarring region containing the VT isthmus site (n=3). Each circle represents an individual bipole. Representative bipoles from each region are represented in green, light red, and blue filled circles, respectively, along with their electrograms. On graphs, values from sample electrodes are marked with triangles. aVL indicates augmented vector left ECG lead.

Another sample case of VT induction with basket‐catheter electrodes located inside the scar tissue and the VT region of interest (based on higher‐resolution sequential activation mapping) is shown in Figure 4A. In this case, the last S1 of the PVS protocol did not show differences in LAT‐DS values between healthy, scar, or VT regions of interest (Figure 4B). However, the S2 of the PVS showed higher LAT‐DS values in the VT region of interest compared with both the rest of the infarcted substrate and healthy myocardium (Figure 4B). Importantly, detection of the VT region of interest was not possible using conventional LAT data with any of the coupled extrastimuli of the PVS protocol.

**Figure 5:**
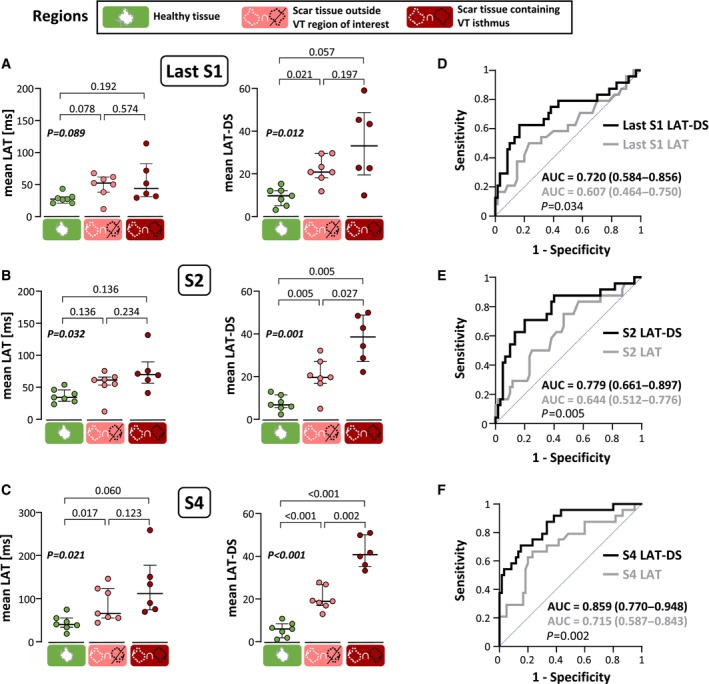
Local activation time‐dispersion score values identify ventricular tachycardia isthmus sites during programed ventricular stimulation. **A** through **C**, Quantification and comparisons of the average local activation times (LAT) and LAT‐dispersion scores (LAT‐DS) during the last S1 (**A**), S2 (**B**), and S4 (**C**) coupled extrastimuli of the programmed ventricular stimulation protocol in myocardial regions inside healthy myocardium, inside a scarring region outside the ventricular tachycardia (VT) region of interest and inside a scarring region containing the VT isthmus. Each animal is represented with 1 circle (Group 1, VT subgroup [N=7]); scarring regions containing VT isthmus sites have 1 missing point (N=6), since in 1 case the VT isthmus site was outside the endocardial area covered with the basket catheter. (**D** through **F)**, Receiver operating characteristic curves comparing the performance of LAT (gray) and LAT‐DS (black) in discerning the myocardial substrate containing the VT isthmus from scarring regions outside the VT isthmus using single‐beat analysis of the last S1 (**D**), S2 (**E**), and S4 (**F**). Data from pigs in Group 1, VT subgroup (N=7). AUC indicates area under the curve.

Overall, single‐beat derived LAT‐DS values during PVS properly identified VT regions of interest containing protected isthmus sites (Figure 5A through 5C). More specifically, during the first (ie, S2) and the last (ie, S4) coupled extrastimuli of the PVS protocol, LAT‐DS values were significantly higher in basket‐catheter electrodes located within VT regions of interest compared with the rest of scar regions (38.6 [27.1–48.8] versus 19.7 [16.8–27.1] for S2, *P*=0.027, Figure 5B; and 41.9 [36.3–51.4] versus 19.7 [17.6–27.7] for S4, *P*=0.002, Figure 5C) and healthy myocardium (38.6 [27.1–48.8] versus 6.8 [5.4–11.4] for S2, *P*=0.005, Figure 5B; and 41.9 [36.3–51.4] versus 6.5 [2.2–8.9] for S4, *P* <0.001, Figure 5C). Conversely, conventional LAT values during the PVS protocol could not differentiate scar regions relevant for VT maintenance during such coupled extrastimuli of the PVS protocol (Figure 5A through 5C). Moreover, receiver operating characteristic curves and area under the curve analyses showed that the LAT‐DS performed significantly better than LAT to discern the myocardial substrate containing the VT isthmus from the rest of the myocardium (0.779 versus 0.644 for S2, *P*=0.005; 0.859 versus 0.715, *P*=0.002 for S4, Figure 5D through 5F). Importantly, the potentially variable distance between neighboring electrodes of the basket catheter (Figure S6) did not significantly affect the performance of the LAT‐DS to identify VT isthmus sites during the PVS protocol (Figure S7). Thus, the area under the curve values of the LAT‐DS were not significantly different from the LAT‐DSsw using any of the alternatives to weight the distance between neighboring electrodes (Figure S7).

### Average Local Activation Time Dispersion Score on Single‐Beat Maps Is Associated With Poststimulation Arrhythmia Severity

Further analyses were performed in animals from Group 2 (N=6), which underwent 46 arrhythmia‐induction attempts using PVS. Poststimulation outcomes were noninducible arrhythmia, sustained monomorphic VT and polymorphic VT/VF in 12, 15, and 19 PVS attempts, respectively (further details are provided in Table S1). A sample case is shown in Figure S8. Overall, the analysis of the last S1 and S2 of the PVS protocol did not show statistical differences on LAT or LAT‐DS values among different poststimulation outcomes (Figure S9A and S9B). Conversely, the S3 and S4 of the PVS protocol showed significantly different values of LAT‐DS when the poststimulation outcome was noninducible arrhythmia, monomorphic VT and polymorphic VT/VF episodes (Figure S9C and S9D). Using the LAT‐DS derived from the S4, the LAT‐DS showed increasingly higher values as the poststimulation arrhythmia severity increased, showing statistical significance in all pairwise comparisons (no induction versus VT: *P*=0.023; no induction versus PVT/VF: *P*=0.009; VT versus PVT/VF: *P*=0.025. Figure S9D). Conversely, mean LAT of S4 beats did not show significant differences among the 3 poststimulation outcomes (Figure S9D). The interested reader can find further details in Data S1.

## DISCUSSION

This experimental study shows that infarct‐related left ventricular endocardial regions with large dispersion of local activation times during PVS are associated with VT isthmus sites during VT mapping. We defined a local activation time dispersion score (LAT‐DS) that, on single‐beat global endocardial maps from basket catheters, can identify regions with higher dispersion of activation times than their neighbors. In animals with comprehensive electrophysiological characterization of reentrant monomorphic VT episodes, we showed that single‐beat analysis of LAT‐DS during the coupled extrastimuli of a PVS protocol enabled identification of infarct‐related regions associated with VT isthmus sites. Further insights from experiments including several PVS attempts in the same animal, showed that LAT‐DS values on single‐beat maps were also related to the poststimulation outcome in terms of arrhythmia severity. Overall, these results prove that a single‐beat strategy during PVS could help to efficiently identify endocardial regions associated with VT isthmus sites in common clinical scenarios as poorly tolerated VT episodes upon VT induction.

Our data emphasize the relevance of identifying functional electrophysiological properties of specific myocardial areas associated with relevant pathways for VT maintenance. Recent studies showed that localized regions with wavefront deceleration can identify VT isthmus sites using sequential high‐resolution maps.^4^, ^5^ Jackson et al further proved the utility of using extrastimuli to evoke decremental conduction before VT initiation.^11^ Indeed, myocardial regions displaying decremental evoked potentials during extrastimuli would facilitate unidirectional conduction block and reentry initiation. Conversely, late potentials without adjacent delayed conduction would not enable to sustain reentry. Identification of these regions has proven to be clinically feasible and their ablation yielded promising outcomes in terms of VT burden reduction.^12^ Our study adds to this approach and shows that heterogeneity of LAT within specific myocardial regions, rather than the absolute values of LAT within such regions (ie, late potentials), is the relevant electrophysiological parameter for detecting highly proarrhythmic infarct‐related areas. Importantly, as compared with a sequential‐mapping approach of only 1 coupled extrastimulus (S2), which indeed requires multiple repetitions of a S1‐S2 pacing protocol,^12^ our single‐beat strategy applies a single run of PVS to identify VT isthmus sites. Moreover, single‐beat‐derived LAT‐DS maps during PVS show a progressive increase in the predictive performance from the S2 to the S4 to identify VT isthmus sites. Our results are also compatible with recent data reporting that repolarization heterogeneity in VT isthmus sites creates the substrate for unidirectional block and reentry intiation.^10^

Single‐beat mapping using a basket catheter in the left ventricle and near‐field electrograms provides some advantages over other approaches to identify the arrhythmogenic substrate associated with infarct‐related VTs. First, in a single beat, well‐distributed electrodes within the endocardial surface are often sufficient to provide accurate and reliable LAT values, comparable to higher‐resolution activation maps during ventricular pacing (Figure S4). This gives multipolar basket catheters the advantage of reporting on a single beat the electrograms recorded over large areas of the endocardial surface. Indeed, a single‐beat map would represent a much faster alternative than current sequential multipolar maps using state‐of‐the‐art tools.^19^ Second, single‐beat maps using near‐field electrograms are not subject to far‐field limitations of body surface potentials to detect small‐amplitude electrograms within the infarct‐related substrate.^20^, ^21^, ^22^ Indeed, low‐amplitude near‐field electrograms are important for detecting LAT at each electrode location and its dispersion score during the coupled extrastimuli of the PVS (Figure 2 and Figure S2). Third, single‐beat maps would enable a rapid analysis of different beats of the PVS protocol without repeating the entire sequential map for each extrastimulus.^5^, ^12^, ^23^, ^24^ Finally, although single‐beat maps from 64‐electrode basket catheters may have limited spatial resolution to identify small protected VT isthmus sites, the spatial resolution (≈5×22 mm^2^, Figure S5A and S5B) may be sufficient to identify endocardial VT isthmuses within the ranges reported in patients with infarct‐related substrate (≈15–38 mm long and ≈9–24 mm wide).^4^, ^9^, ^25^ Nevertheless, this limited spatial resolution would benefit from the development of new basket catheters with more electrodes (eg, 128 electrodes and including distal electrodes to cover the apex), which would be compatible with the most common electroanatomical mapping systems used in the clinic. Moreover, patients suspected of not benefiting from commercially available basket catheter designs (ie, with apical and/or epicardial VT circuits) could be screened beforehand using CMR imaging, electrophysiological data, or both.^26^, ^27^, ^28^

This study has some limitations. First, although single‐beat analysis of LAT‐DS identified infarct‐related regions associated with endocardial VT isthmus sites, this was only validated in a subgroup of animals with well‐tolerated VT episodes. Whether the induction of multiple VT morphologies will present different high LAT‐DS regions ought to be addressed. Second, radiofrequency energy delivery in the pig ventricle often yields VF.^29^ Therefore, the implications on ventricular arrhythmia inducibility after the ablation of ventricular regions with high LAT‐DS values could not be addressed in our experimental model. Third, basket catheters like the one used in this study are not routinely used in conventional electrophysiology procedures, although such catheters have been previously reported in clinical research series.^30^ Indeed, catheter positioning inside the left ventricle was performed easily and without complications in the pig model. Importantly, the LAT‐DS analysis would be adaptable to nonglobal mapping approaches using large multipolar catheters with a fixed electrode array structure (eg, Advisor HD Grid Mapping Catheter, Abbott; or OPTRELL Mapping Catheter, Biosense Webster). The latter would require a more time‐consuming mapping procedure, although it would improve spatial resolution and could benefit from novel technologies such as omnipolar mapping and automatic near‐field detection.^31^, ^32^ Notwithstanding, the optimal definition of the LAT‐DS, depending on the mapping methodology used, ought to be addressed in further studies, although the one presented in this study should be optimal for state‐of‐the‐art catheters with a fixed electrode array structure.

## CONCLUSIONS

LAT‐DS analysis of single‐beat global endocardial maps during PVS enables identification of infarct‐related regions with highly heterogeneous activation times that are associated with protected VT isthmus sites.

## Sources of Funding

The Centro Nacional de Investigaciones Cardiovasculares (CNIC) is supported by the Ministry of Science, Innovation and Universities (MCIU) and the Pro CNIC Foundation, and is a Severo Ochoa Center of Excellence (CEX2020‐001041‐S). This study was also supported by grants from the Heart Rhythm Association of the Spanish Society of Cardiology (2020), the Fondo Europeo de Desarrollo Regional (CB16/11/00458), the MCIU (PID2019‐109329RB‐I00), and the Comunidad de Madrid (S2022/BMD‐7229, Arcadia). The study was partially supported by the Fundación Interhospitalaria para la Investigación Cardiovascular (FIC, Madrid, Spain).

## Disclosures

None.

## Supporting information

Data S1
Table S1
Figures S1–S9
