# Supplementary material for: Dispersion of Activation in Single‐Beat Global Maps During Programmed Ventricular Stimulation Identifies Infarct‐Related Ventricular Tachycardia Isthmus Sites
Source: J Am Heart Assoc. 2024 Nov 22;13(23):e038441. doi: 10.1161/JAHA.124.038441 (PMC11681573; doi:10.1161/JAHA.124.038441)
Supplement: Supplementary file 1 — Data S1 Table S1 Figures S1–S9 [file JAH3-13-e038441-s001.pdf]

Supplemental Material

Data S1.

Supplemental Methods

Anesthesia protocol

All procedures in pigs were performed under general anesthesia. Anesthesia induction was achieved by intramuscular ketamine injection (15 mg/kg), xylazine (2 mg/kg) and midazolam (0.5 mg/kg). Then, the pigs were intubated and mechanically ventilated with oxygen (fraction of inspired O_2 of 21%) and anesthesia was maintained with continuous intravenous infusion of ketamine (2 mg/kg/h), xylazine (0.2 mg/kg/h) and midazolam (0.2 mg/kg/h). Unfractionated heparin (300 UI/kg) was administered at the onset of instrumentation during invasive procedures. The femoral artery was also used for continuous monitoring of blood pressure during electrophysiology studies.

Pig model of myocardial infarction

Pigs underwent percutaneous catheterization of the left anterior descending (LAD) coronary artery to inflate an angioplasty balloon and occlude the artery for 60 minutes. The balloon was inflated either proximal or distal to the first diagonal branch to generate different infarct sizes and variable scar distributions. A continuous infusion of intravenous amiodarone (150 mg/h) was administered to decrease the incidence of malignant arrhythmias. In case of ventricular fibrillation (VF) during the ischemia protocol, a non-synchronized shock was delivered with a biphasic defibrillator. After 60 minutes of occlusion, the balloon was deflated and a coronary angiogram was recorded to confirm patency of the coronary artery and reperfusion.

Multi-induction programmed stimulation protocol to assess poststimulation arrhythmia severity

In pigs from Group 2 (N=6), the electrophysiological study included PVS with a minimum of 5 inducibility attempts to assess heterogeneity on single-beat activation patterns and their association with poststimulation outcomes, which were classified as non-inducible arrhythmia, sustained monomorphic ventricular tachycardia (VT) or polymorphic VT/VF. After a minimum of 30 seconds in monomorphic VT (if hemodynamically tolerated) the arrhythmia was terminated with overdrive pacing or electrical cardioversion synchronized with the intrinsic QRS complex. If induction attempts yielded polymorphic VT/VF with hemodynamic collapse, the arrhythmia was terminated with a biphasic DC shock (200 Joules). Animals were allowed to recover for at least 10 minutes after termination of any ventricular arrhythmia episode. Continuous monitoring of the 12-lead ECG and invasive blood pressure were used to ensure recovery to baseline conditions after VT/VF termination and before a new inducibility attempt. A schematic of the protocol is shown in Figure S8A and further details of the induced arrhythmias for each animal are provided in Table S1.

For the location of the basket catheter, X-ray images were recaptured after DC shocks to ensure location stability before subsequent PVS within the protocol. High-density geometries using an electroanatomical mapping system were not acquired, and therefore, electrodes positions were directly projected onto the CMR geometry after registration (see following sections).

Projection of late gadolinium enhancement cardiac magnetic resonance (LGE-CMR) imaging-derived scar onto electroanatomical mapping geometries

In animals from Group 1, 3D LGE-CMR geometries were registered to their corresponding electroanatomical mapping geometries. The aortic root, the mitral valve and left ventricular apex were used as anatomical landmarks before registration using a custom-made software tool in Matlab (MathWorks Inc., Natick, MA). Then, an iterative closest-point algorithm was applied to the resulting surfaces to project the LGE-CMR derived scar onto the electroanatomical mapping geometry.

Catheter location and projection onto LGE-CMR or electroanatomical mapping derived geometries

Catheter electrodes were identified and tagged, and the heart contour was delineated in orthogonal fluoroscopic images acquired during the electrophysiology study. Each spline of the basket catheter (A, B, C, D, …) was identified based on a specific marker based on the position of a wider electrode within the spline, which is visible in X-ray images. Electroanatomical mapping geometries (in Group 1) or LGE-CMR (in Group 2) geometries were registered onto the fluoroscopic images using their contours in the corresponding orthogonal views (antero-posterior and left-lateral). Electrodes displaying atrial and ventricular electrograms with similar amplitude guided the delineation of annular regions. Electrodes were then projected onto the geometries using a minimum-total-distance criterion (adding distances from both views). Electrodes displaying poor contact with overt far field signal were discarded and those projected in an inconsistent manner (i.e., not following the basket catheter shape) were manually modified. Electrodes projected into LGE-CMR-derived scar regions were confirmed by the presence of fragmented potentials. Figure S1 shows the complete process.

Comparison of basket-catheter derived single-beat maps and higher resolution sequential activation maps

In animals from Group 1, basket-catheter derived single-beat activation maps during S1 pacing at 500 ms cycle length were compared with higher-density sequential activation maps. For each basket-catheter electrode, local activation times (LAT) from 5 consecutive beats were averaged and compared with the closest mesh point LAT on the higher resolution activation map. Differences were computed for each basket-catheter electrode and averaged to obtain mean absolute and relative (to the high-density map total activation time) errors (MAE and MRE, respectively). Since single-beat and higher resolution maps had different ECG references (registered with the Labsystem PRO EP recording system and Carto3 system, respectively), references were offset to enable comparisons. The Passing-Bablok regression method was used to test the linear

relationship between them. A linear relationship can be assumed if the confidence intervals of the slope and the intercept include the values of 1 and 0, respectively (Figure S4E).

Calculation of local activation time-dispersion score

For every electrode in the basket catheter, the local activation time-dispersion score (LAT-DS) was calculated as follows:

$$LATDS = \frac{LAT \cdot LAT_{SD}}{AT_{total}^2} \cdot 200$$

where *LAT* is the electrode local activation time, LAT_{SD} is the standard deviation of the LAT in neighboring electrodes, and AT_{total} is the total activation time. The resulting value yields a unitless score. A multiplying factor of 200 is applied to draw a final score ranging from 0 to 100.

Neighbor electrodes are defined as those immediately distal and proximal in the same and in adjacent splines (i.e., all electrodes have 8 neighbors, except for distal and proximal electrodes in each spline that have 5). Total activation time is calculated as earliest-to-latest LAT, including right ventricular activation time when available. A representative example is shown in Figure S2.

Calculation of spatially-weighted local activation time-dispersion score

In order to account for the varying distance between neighboring electrodes, a spatially-weighted variant for the LAT-DS was also tested (i.e., LAT-DSsw) and calculated as follows:

$$LATDS_{sw}(i) = \frac{LAT(i) \cdot LAT_{SD_w}(i)}{AT_{total}^2} \cdot 200$$

All factors in the formula are the same except for the standard deviation of the LAT, that is substituted by the weighted standard deviation (LAT_{SDw}). In order to calculate LAT_{SDw}, each neighboring electrode LAT (defined the same way as in the original LAT-DS) will have a varying weight ($w_{i,j}$) inversely proportional to the distance to the analyzed electrode:

$$LAT_{SD_w}(i) = \sqrt{\frac{\sum_{j=1}^{N} w_{i,j} \, |LAT(j) - \overline{LAT_w}|^2}{\sum_{j=1}^{N} w_{i,j}}} \qquad w_{i,j} = \left(\frac{d_{min}}{d_{i,j}}\right)^a$$

$\overline{LAT_w}$ is the weighted average LAT of all neighbors and $d_{i,j}$ is the distance from the neighbor electrode *j* to the analyzed electrode *i*. The weight is normalized to the distance to the closest neighbor, d_{min}, which will typically be the fixed inter-electrode distance within a spline (~5 mm). Finally, the weight is modulated by an exponentiation factor *a* to adjust the "severity" of an increasing distance in the resulting weight. Note a value of *a=0* would result in a weight factor of 1 for all neighboring electrodes (i.e., *LATDS =*

$LATDS_{sw}$), and an extremely high value of *a* would cancel the effect of more distant electrodes in adjacent splines in the activation time dispersion analysis.

A schematic of the complete process for LAT-DSsw calculation is shown in Figure S3A and a sample case with LAT-DSsw maps for different values of the weighting factor *a* is shown in Figure S3B.

Extended Results

Effect of varying neighboring distance on the LAT-DS analysis

A sample case from Group 1, VT subgroup, showing the average distances to neighbors is presented in Figure S6A. In this case, the electrodes showing the highest LAT-DS values (located inside the VT region of interest) during the first coupled extrastimulus (S2) did not show the highest average distance to its neighbors. The same analysis in all animals from the VT subgroup (Group 1) showed that there was no significant correlation between the average or maximum distances to neighbors and their resulting LAT-DS values, when analyzing the first (S2) and third (S4) coupled extrastimuli of the pacing protocol (Figure S6B, C, respectively). Furthermore, there were no statistically significant differences in the mean and maximum distances to neighbors between electrodes inside regions of healthy tissue, scar tissue outside the VT region of interest or the scar tissue containing the VT isthmus (Figure S6D, E, respectively).

Extended results on the average LAT-DS values associated with poststimulation arrhythmia severity

A sample case with 3 representative poststimulation outcomes (non-inducible arrhythmia, sustained monomorphic VT and polymorphic VT/VF) is shown in Figure S8B. In this specific case, during the S4 of the PVS protocol, electrodes located inside the infarct-related scar region (based on LGE-CMR images) showed higher values of LAT-DS than those located in healthy tissue (light-red vs. green dots in Figure S8B). In the same animal, VF induction after the S4 of the PVS protocol was associated with higher average values of LAT-DS than the same protocol when the outcome was sustained monomorphic VT or non-inducible arrhythmia (18.6 vs. 15.6 and 12.4, respectively (Figure S8B).

Overall, in animals from Group 2, the analysis of the last S1 and S2 of the PVS protocol did not show statistically significant different values of LAT or LAT-DS among different poststimulation outcomes (Figure S9A, B). However, the S3 and S4 of the PVS protocol did show significantly different values of LAT-DS when the poststimulation outcome was non-inducible arrhythmia, monomorphic VT and polymorphic VT/VF episodes (12.4 [9.9, 14.3] vs. 12.4 [10.2, 15.1] vs. 16.0 [14.0, 18.0], p=0.005 in S3, Figure S9C; and 12.0 [10.4, 12.4] vs. 14.2 [13.8, 15.2] vs. 15.7 [14.6, 16.6], p=0.006 in S4, Figure S9D). Indeed, the LAT-DS values of the S4 were increasingly higher as the poststimulation arrhythmia severity increased, showing statistical significance in all pairwise comparisons (no

induction vs. VT: p=0.023; no induction vs. PVT/VF: p=0.009; VT vs. PVT/VF: p=0.025, Figure S9D). Conversely, mean LAT of S3/S4 beats did not show significant differences among the 3 poststimulation outcomes (Figure S9C, D, leftmost columns).

In further analyses, electrodes located within scar regions were compared to those in healthy tissue (Figure S9 E-H). During the second and third extrastimuli of PVS protocol (S3 and S4, respectively), LAT-DS values were significantly higher in electrodes located within scar regions compared to those in healthy tissue (14.7 [14.2, 18.3] vs. 10.9 [8.7, 15.3], p=0.054 in S3, Figure S9G; and 15.7 [15.4, 16.9] vs. 11.2 [9.8, 12.7], p=0.012 in S4, Figure S9H), regardless of the poststimulation outcome. The latter is consistent with the higher arrhythmogenicity of the slow-conducting tissue within scar regions compared to the rest of the myocardium.

Table S1. Multi-induction protocol data (Group 2)

	No induction	VT	PVT/VF
**Animal 1**			
n (%)	1 (20.0)	0 (0)	4 (80.0)
3 coupled extrastimuli	0	-	2
4 coupled extrastimuli	1	-	2
**Animal 2**			
n (%)	5 (50.0)	4 (40.0)	1 (10.0)
3 coupled extrastimuli	0	0	0
4 coupled extrastimuli	5	4	1
**Animal 3**			
n (%)	1 (12.5)	5 (62.5)	2 (25.0)
3 coupled extrastimuli	0	0	1
4 coupled extrastimuli	1	5	1
**Animal 4**			
n (%)	1 (11.1)	3 (33.3)	5 (55.5)
3 coupled extrastimuli	0	1	1
4 coupled extrastimuli	1	2	4
**Animal 5**			
n (%)	3 (37.5)	2 (25.0)	3 (37.5)
3 coupled extrastimuli	0	0	0
4 coupled extrastimuli	3	2	3
**Animal 6**			
n (%)	1 (16.6)	1 (16.6)	4 (66.6)
3 coupled extrastimuli	1	0	1
4 coupled extrastimuli	0	1	3
**Total**			
n (%)	12 (26.0)	15 (32.6)	19 (41.3)
3 coupled extrastimuli (%)	1 (8.33)	1 (6.66)	5 (26.3)
4 coupled extrastimuli (%)	11 (91.6)	14 (93.3)	14 (73.6)
BDCL (ms)	280 (270, 342.5)	280 (280, 300)	300 (280, 300)
S3 coupling interval (ms)	195 (182.5, 220)	200 (180, 220)	190 (180, 200)
S4 coupling interval (ms)	190 (180, 217.5)	190 (180, 190)	180 (180, 190)

Values are expressed as median and interquartile ranges and n (%), as appropriate. BDCL: basic drive cycle length. LAT-DS: local activation time-dispersion score. PVT: polymorphic ventricular tachycardia. VF: ventricular fibrillation. VT: ventricular tachycardia

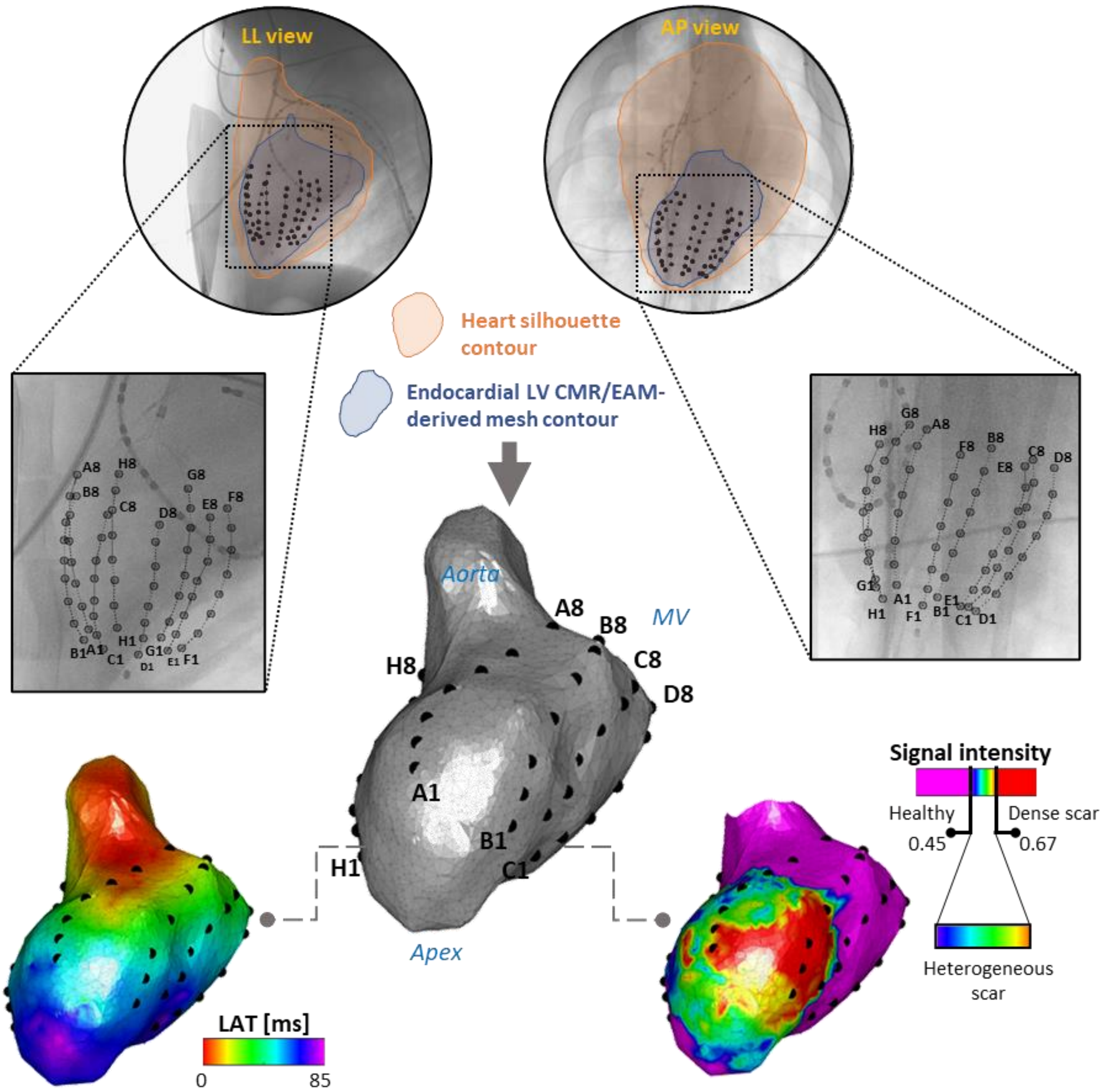

Figure S1. Schematic workflow of basket catheter location on the left ventricular space. Top row, sample orthogonal heart views (Left lateral [LL] and anteroposterior [AP] views) with basket-catheter visualization into the left ventricular endocardial mesh from cardiac magnetic resonance (CMR) imaging or the electroanatomical map (EAM). Manually-delineated heart contours on the X-ray views are highlighted in orange. Left ventricular endocardial contour is highlighted in blue. Basket-catheter electrodes are highlighted with black dots and linearly connected intra-spline. A zoom-in of the basket electrode is depicted for both the LL and the AP views, tagging proximal and distal electrodes. Central illustration and bottom row show a representative basket catheter positioning and electrode visualization on the left ventricular CMR/EAM surface. Visible electrodes on the AP view are highlighted with black dots. Distal and proximal electrodes from visible splines are tagged. Bottom left, sample sequential high-resolution activation map with concomitant visualization of basket-catheter electrodes onto the left ventricular endocardial electroanatomical mesh. Bottom right, sample late-gadolinium-enhancement CMR imaging projected onto the electroanatomical mesh of the left ventricle using a 0.45 and 0.67 normalized signal intensity criteria for heterogeneous and dense scar, respectively, and further visualization of basket-catheter electrodes.

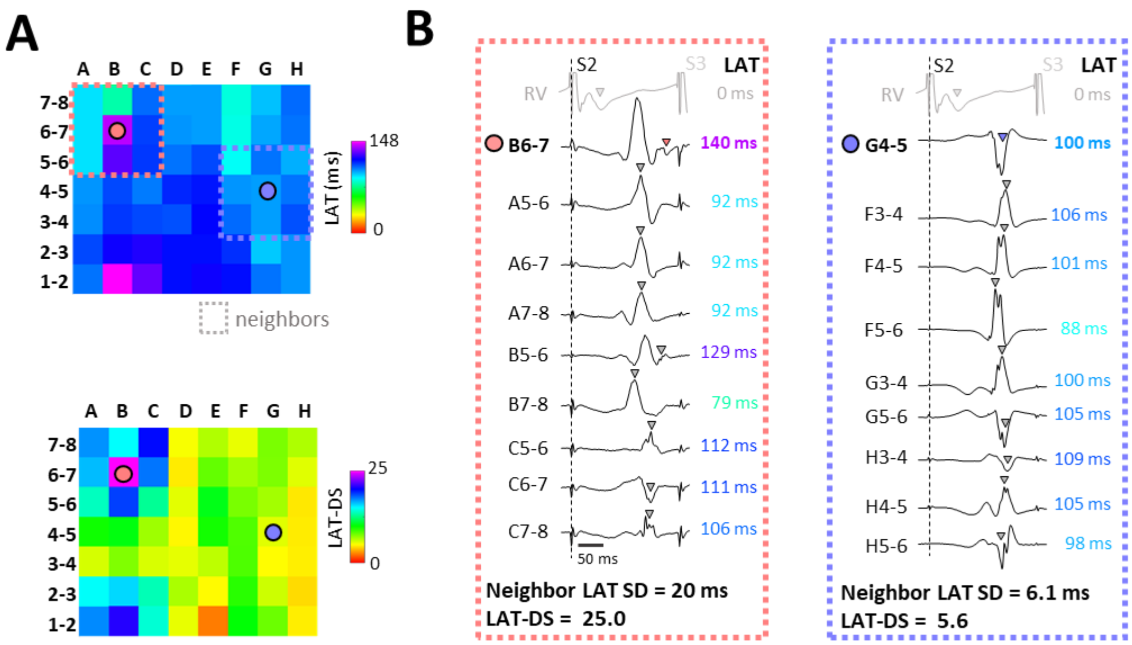

Figure S2. Single-beat local activation time-dispersion score analysis during programmed ventricular stimulation. A, Two-dimensional representation of a basket catheter-derived single-beat local activation time (LAT) map (top row) and local activation time-dispersion score (LAT-DS) map (bottom row) from the first coupled extrastimuli (S2) of the programmed ventricular stimulation protocol (sample case from Figure 2). Electrodes are labelled with a letter to define the spline (A-to-H) and a number to define the intra-spline bipole from distal (1-2) to proximal (7-8). Two representative bipoles from the basket catheter are highlighted with colored circles (light-red and blue). Neighboring electrodes for each representative bipole, defined as those at immediate adjacent poles and splines, are bounded with dashed lines in the LAT map. **B,** Electrograms from the two bipoles highlighted in light-red and blue (left and right, respectively) and its neighboring bipoles. Local activation times for each electrogram are indicated with a triangle. Top electrogram in grey is from an electrode in the right ventricle (RV) close to the stimulation site, which was used as reference for the total activation time. LAT values are expressed alongside the electrogram and color-coded according to the color-scale on the LAT map (top row in **A**). Resulting neighbor LAT standard deviation (SD) and the LAT-DS are expressed below electrograms.

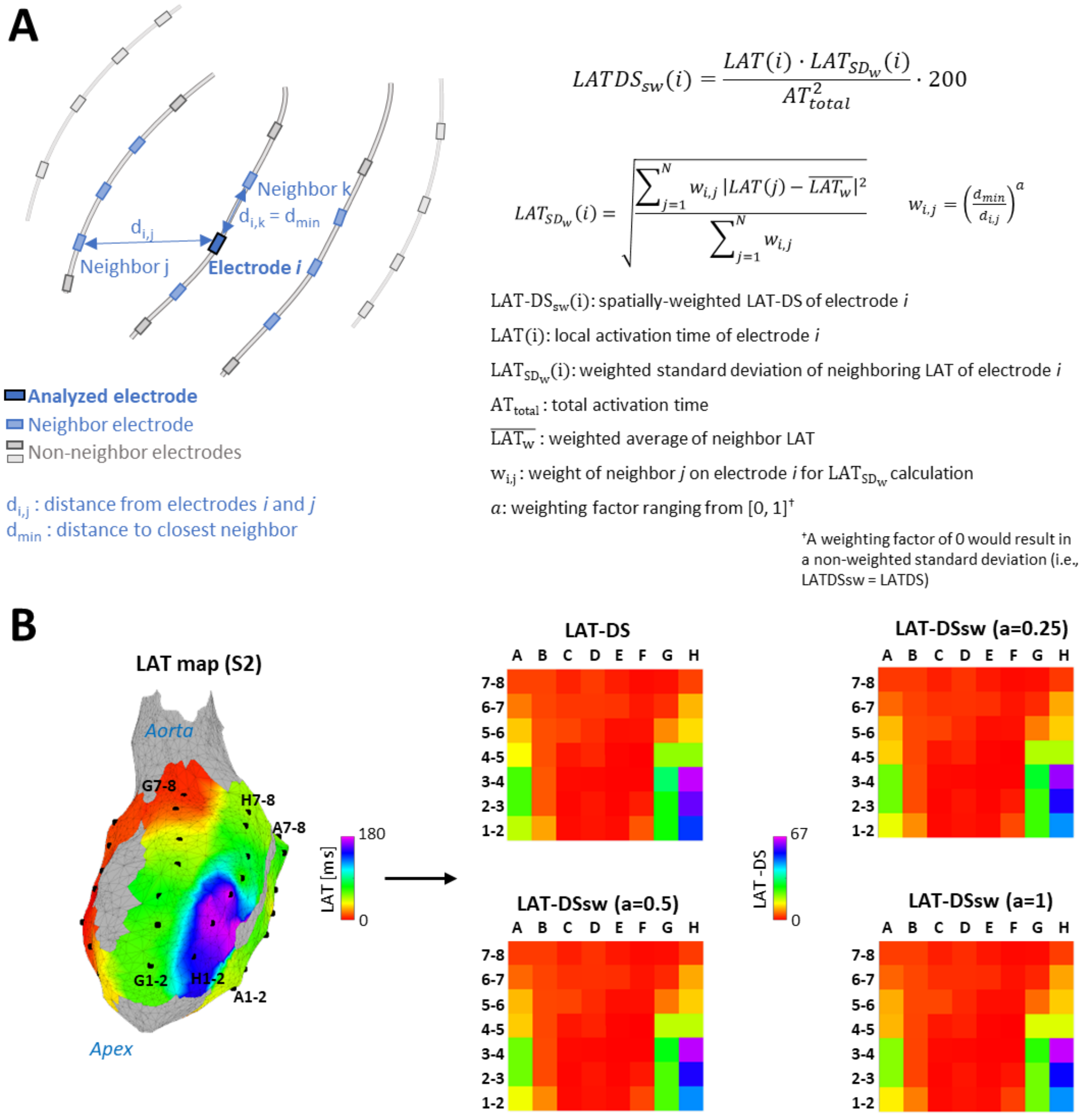

$$LATDS_{sw}(i) = \frac{LAT(i) \cdot LAT_{SD_w}(i)}{AT_{total}^2} \cdot 200$$

$$LAT_{SD_w}(i) = \sqrt{\frac{\sum_{j=1}^{N} w_{i,j}\,|LAT(j) - \overline{LAT_w}|^2}{\sum_{j=1}^{N} w_{i,j}}} \qquad w_{i,j} = \left(\frac{d_{min}}{d_{i,j}}\right)^a$$

Figure S3. Analysis of the local activation time-dispersion score spatially weighted by the distance to neighboring electrodes. A, Schematic and formulas for the calculation of the local activation-dispersion score (LAT-DS) spatially weighted by the distance to neighboring electrodes (LAT-DSsw). Briefly, the LAT-DSsw was calculated as the product of its LAT and the neighboring weighted standard deviation (SD_w) of the LATs, normalized to the squared total single-beat activation time. The weight of each neighboring electrode is inversely proportional to the distance to the analyzed electrode and raised to the power of the modulating factor *a*. **B,** Sample case showing the 3D LAT map (left) and 2D LAT-DS and LAT-DSsw maps (center to right) for different values of the weighting factor *a*. The sample case is the same as in Figure 3A.

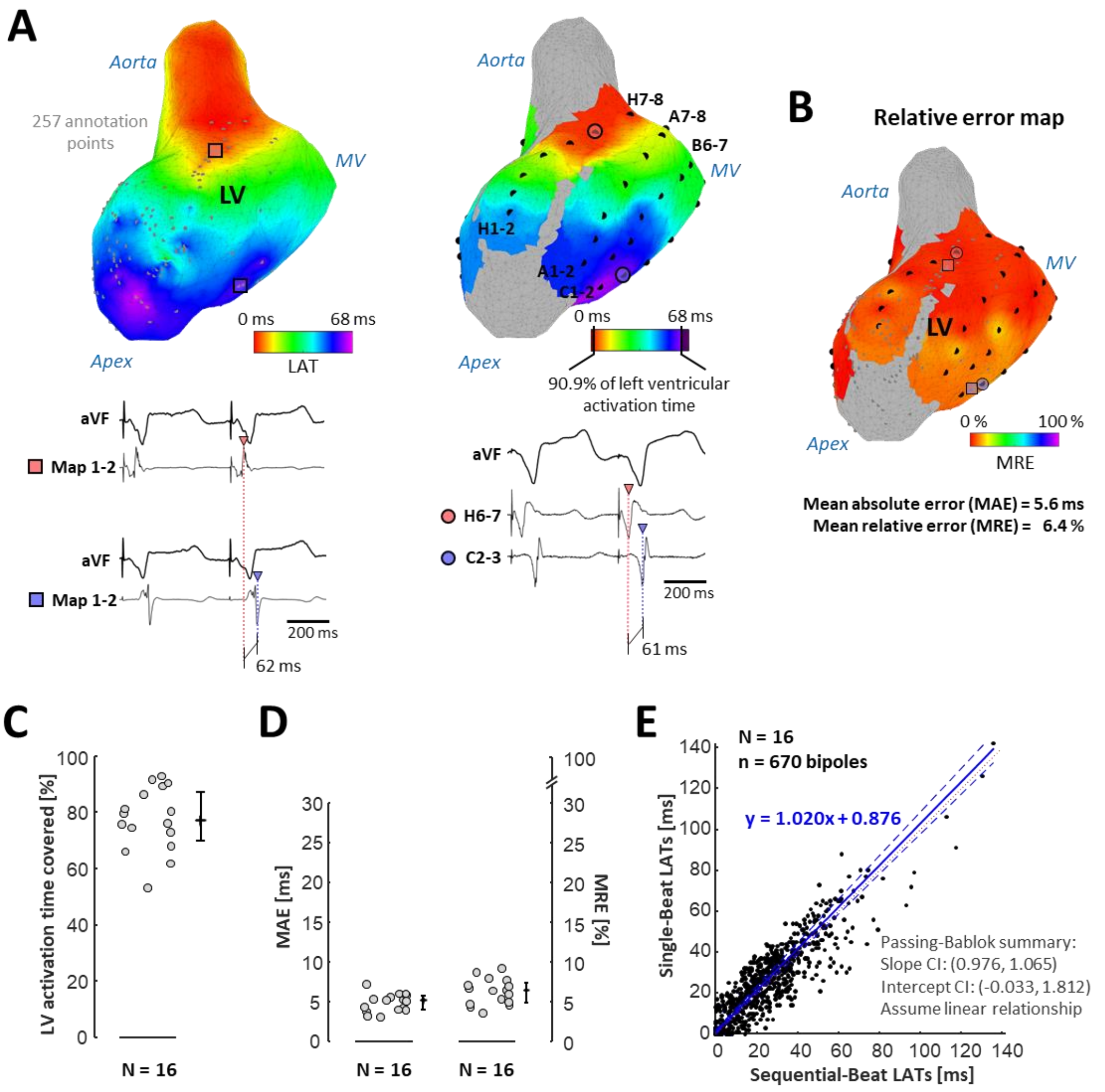

Figure S4. Correlation of single-beat basket-catheter derived activation maps with sequential higher resolution maps. A, Sample comparison of a local activation time (LAT) map during ventricular pacing at 500 ms basic drive cycle length from the right ventricle acquired with sequential point-by-point electroanatomical mapping on the Carto3 system (left), and the equivalent single-beat LAT map using the 64-electrode basket catheter (right). Basket catheter positioning in the left ventricular (LV) mesh of the Carto system was based on localization of basket-catheter electrodes on orthogonal X-ray views and further registration with left ventricular electroanatomical mesh (further details are provided in Figure S1). Squares show sample annotation points on the sequential point-by-point map and circles show sample annotation points on the basket catheter. Basket-catheter electrodes for each of the visible splines on the AP view are represented as black dots. Proximal and distal bipoles are tagged. Sample electrograms of early and late activation sites referenced to the surface aVF lead are shown below each map (red and blue, respectively). **B,** Relative error map, and computed mean absolute and relative errors (MAE and MRE, respectively) of the single-beat LAT map compared to sample annotation points at similar locations. **C,** Quantification of the single-beat activation time covered by the basket catheter as a proportion of the total LV activation time documented in higher-density sequential activation maps during S1 pacing at 500 ms cycle length in animals from Group 1 (N=16). **D,** Quantification of MAE and MRE (left and right, respectively) of single-beat activation maps during S1 pacing at 500 ms cycle length compared with higher-density sequential activation maps. **E,** Passing-Bablok regression of sequential versus single-beat LAT values. The value of 1 is within the confidence interval (CI) of the slope, and the value of 0 is within the CI of the intercept, therefore a linear relationship can be assumed. MV: mitral valve.

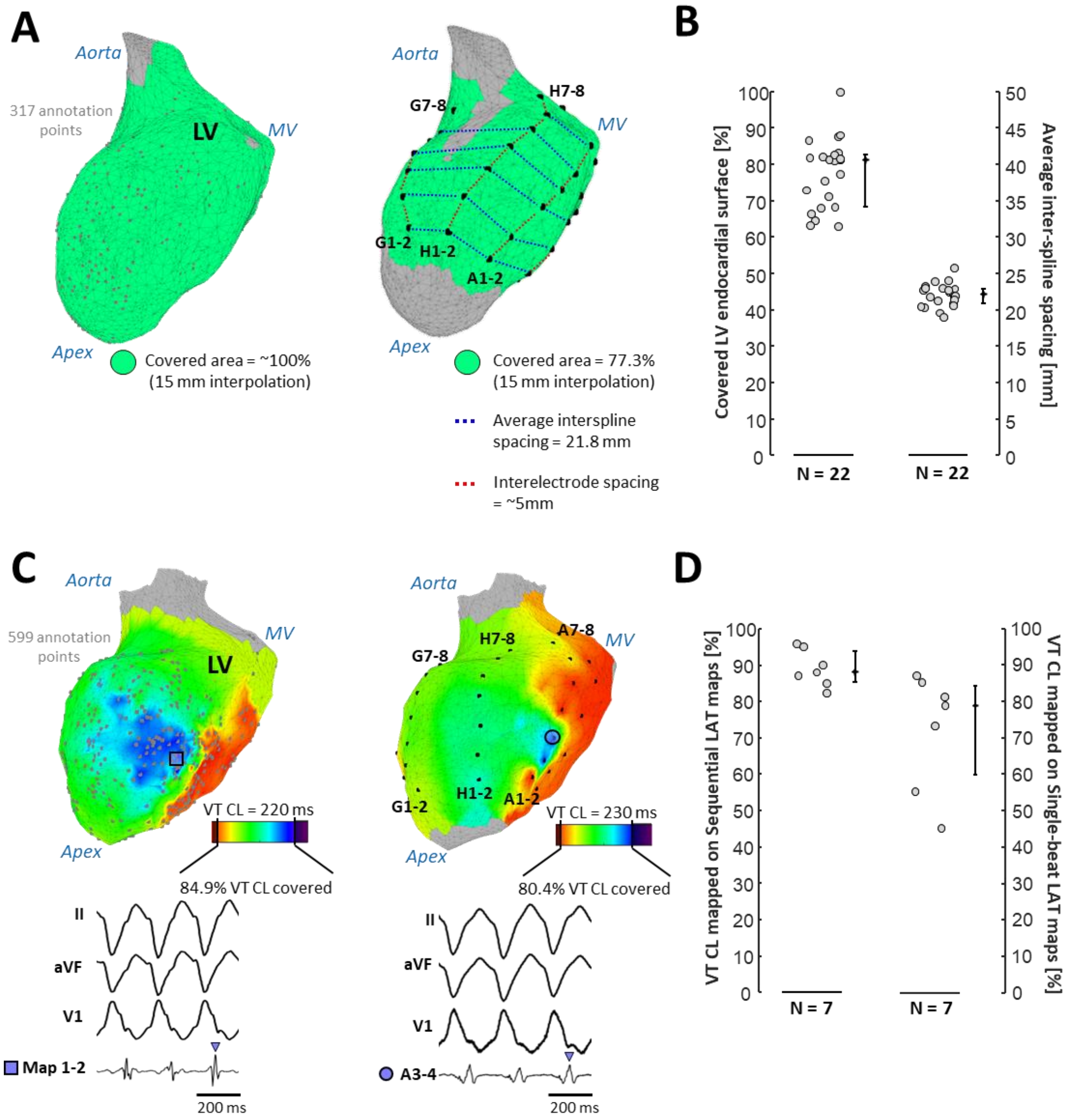

Figure S5. Single-beat basket catheter for the analysis of activation patterns during pacing or ventricular tachycardia. A, Sample comparison of the left ventricular (LV) endocardial surface covered with sequential activation mapping (left) and with a single-beat 64-electrode basket catheter (right). Geometries were acquired from the electroanatomical mapping Carto3 system. Basket catheter positioning was based on the methodology provided in Figure S1. Areas covered with an interpolation of 15 mm are colored in green. Basket-catheter electrodes for each of the visible splines on the AP view are represented as black dots. Visible proximal and distal electrodes are tagged. Basket-catheter interelectrode spacing within a spline and interspline spacing are highlighted with dotted lines (red and blue, respectively). **B,** Quantification of the LV endocardial surface covered with the basket catheter (left) and the average interspline spacing (right) in all animals (N=22). **C,** Sample comparison of a local activation time (LAT) map during ventricular tachycardia (VT) acquired with sequential point-by-point electroanatomical mapping, and a single-beat LAT map from the 64-electrode basket catheter (right). Sample electrograms of late activation sites and representative ECG leads (II, aVF and V1) are shown below each map. The percentage of the VT cycle length (CL) covered by LAT annotations on each map is shown for each map. **D,** Quantification of the percentage of the VT CL covered by LAT annotations acquired with sequential point-by-point electroanatomical mapping (left) and single-beat mapping with the basket catheter (right) in animals from Group 1, VT subgroup (N=7).

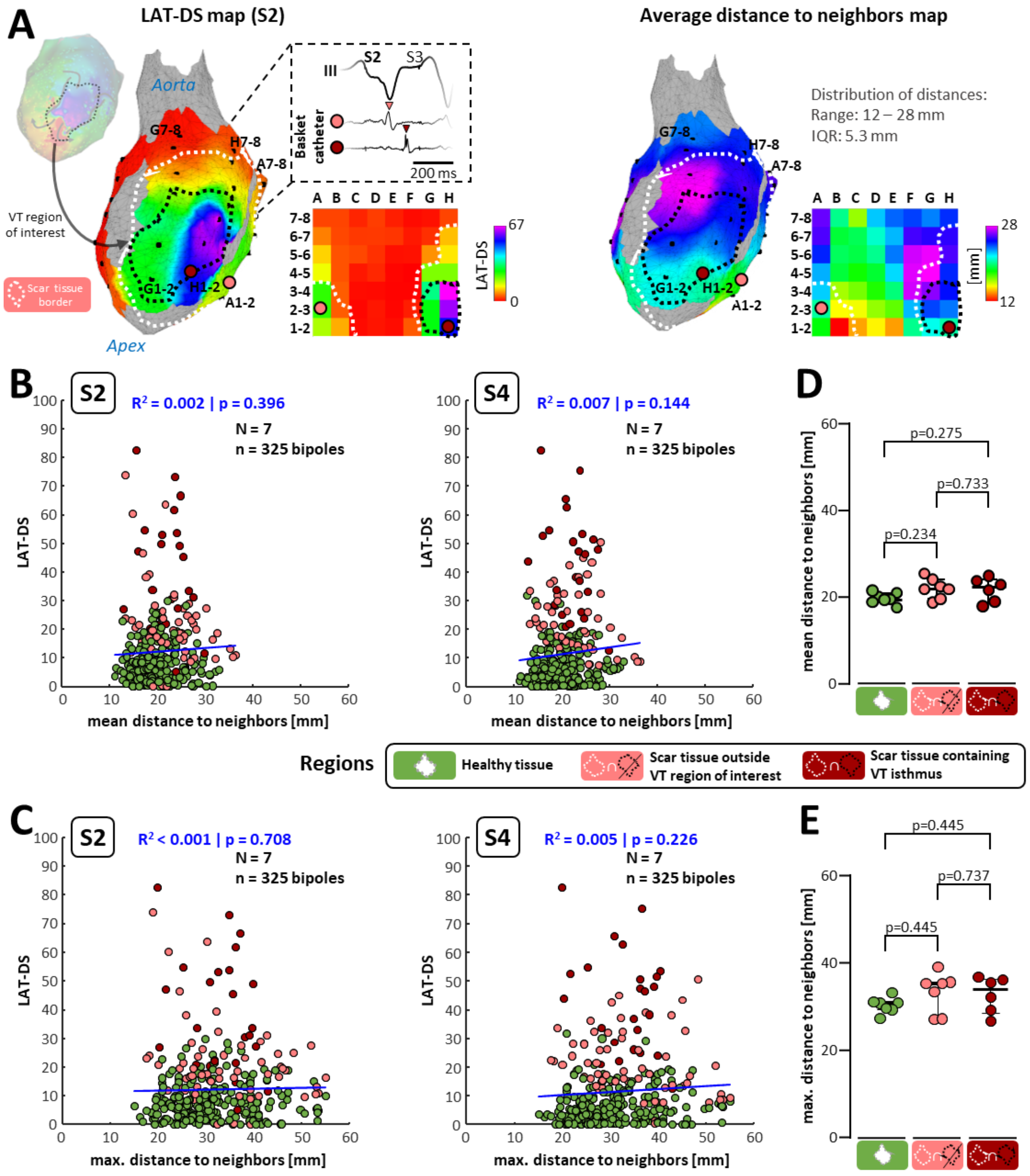

Figure S6. Dependance of the distance to neighboring electrodes on the local activation time-dispersion score values. A, Left, sample case showing 3D and 2D (bottom right) local activation time-dispersion score (LAT-DS) map of the first coupled extrastimulus (S2) during the programmed stimulation (PVS) protocol. The case corresponds to the sample case shown in Figure 3A (see the ventricular tachycardia [VT] activation map shaded on the top left corner). The VT region of interest (containing the isthmus site) is defined using the +10% to -35% of VT cycle length (VT CL) and is represented with a black dashed line. The reference for assigning activation times during VT was the QRS onset. Colored circles indicate sample activation points from scarring regions inside and outside the VT region of interest. Black dots indicate basket-catheter electrodes on each of the visible splines. Right, 3D and 2D (bottom right) maps of the average distance to neighbors. **B, C,** Correlation between local activation time-dispersion score (LAT-DS) values of the S2 (left) and S4 (right) of the PVS protocol and mean **(B)** and maximum **(C)** distances from each electrode to its neighbors. Each circle represents an electrode. Electrodes are color-coded according to the regions of the legend: inside healthy myocardium (green), inside a scarring region outside the VT region of interest (light-red) and inside a scarring region containing the VT isthmus site (dark-red). Data from pigs in Group 1, VT subgroup (N=7). **D, E,** Quantification and comparisons of the average mean **(D)**

and maximum **(E)** distance-to-neighbor values in the same regions. Each animal is represented with one circle (Group 1, VT subgroup [N=7]); scarring regions containing the VT isthmus have one missing point (N=6) as in one case the basket catheter did not include electrodes inside the identified VT isthmus site.

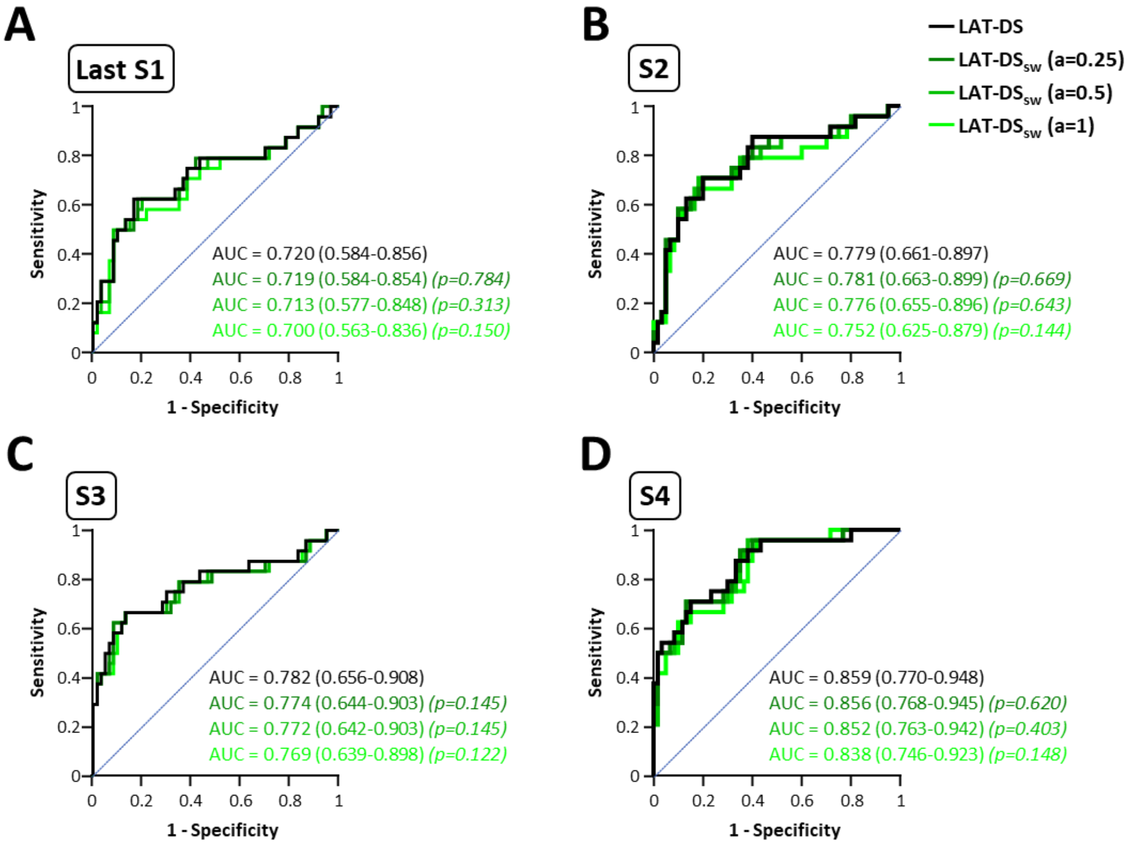

Figure S7. Performance of the local activation time-dispersion score spatially-weighted by the distance to neighboring electrodes. A-D, Receiver operating characteristic (ROC) curves comparing the performance of LAT-DS (black) and LAT-DSsw with different values of the weighting factor *a* (shades of green) in discerning the myocardial substrate containing the VT isthmus from scarring regions outside the VT isthmus using single-beat analysis of the last S1 **(A)**, S2 **(B)**, S3 **(C)** and S4 **(D)**. *p* values show the statistical comparison between each LAT-DSsw against the LAT-DS. Data from pigs in Group 1, VT subgroup (N=7). AUC: area under the curve.

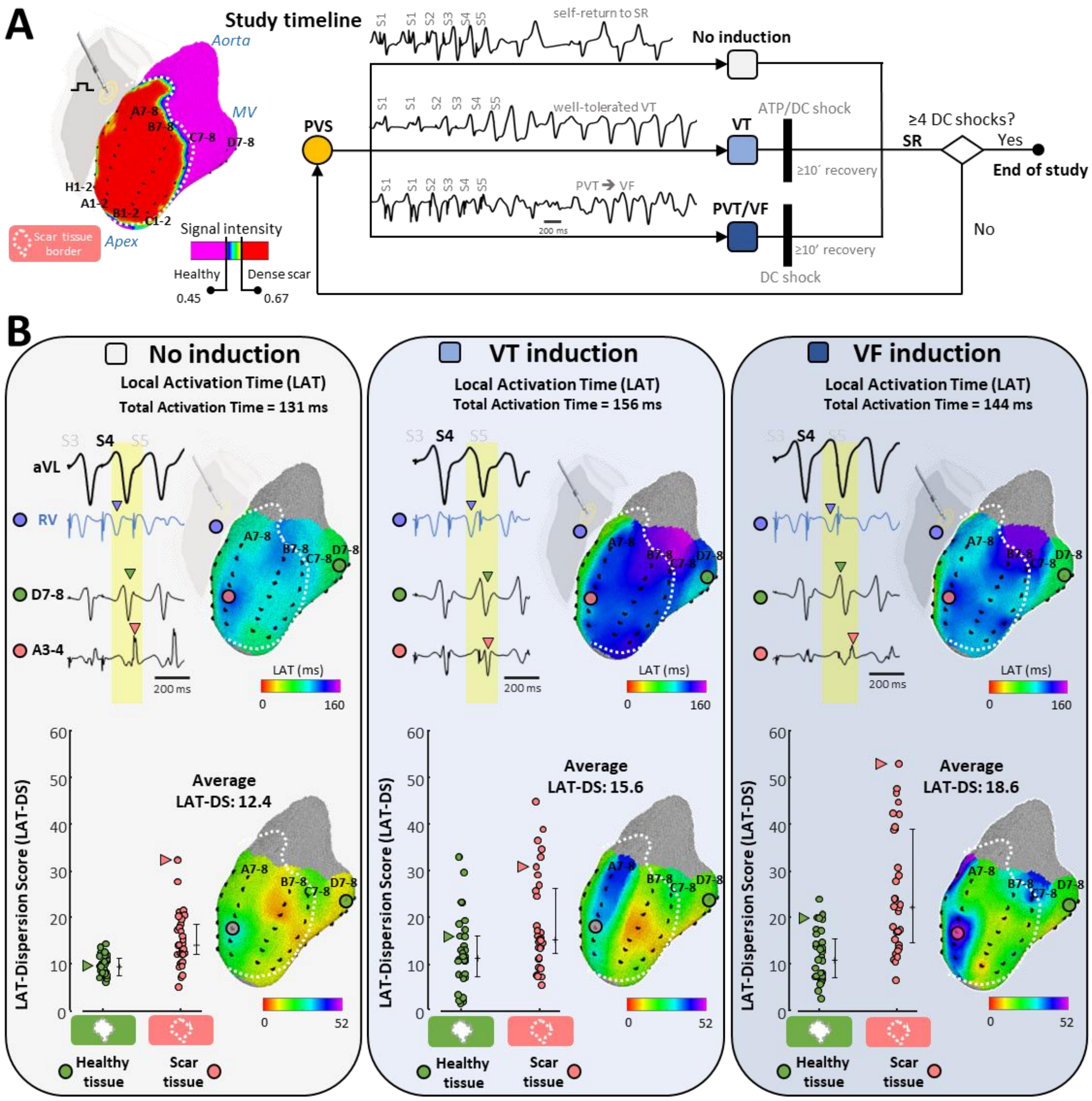

Figure S8. Average local activation time-dispersion score determines the severity of poststimulation outcome. A, Schematic of the experimental protocol in pigs from Group 2. **B,** Sample comparison of local activation times (LAT) (top row) and LAT-dispersion score (LAT-DS) values (bottom row) of the S4 coupled interval (180 ms coupling) of 3 different induction attempts in the same heart that yielded no induction of arrhythmia (leftmost column), monomorphic ventricular tachycardia (VT) (middle column) and ventricular fibrillation (VF) (rightmost column). Blue, green and light red filled circles indicate electrograms from a bipole close to the pacing site in the right ventricle (used as earliest activation time reference), a basket-catheter bipole in a healthy myocardial region, and a basket-catheter bipole from a scarring region, respectively. The displayed electrograms from the scarring region (light red circle) show different morphologies for each poststimulation outcome, which suggests different propagation patterns in that region that may be related to the arrhythmia outcome. The scar contour is represented with a white dashed line. On bottom graphs, values from sample electrodes are indicated with triangles. Each circle represents an individual bipole (n=28 inside the healthy region, n=28 inside the scar region). ATP: antitachycardia pacing; DC shock: direct current shock; MV: mitral valve; PVT: polymorphic VT; SR: sinus rhythm.

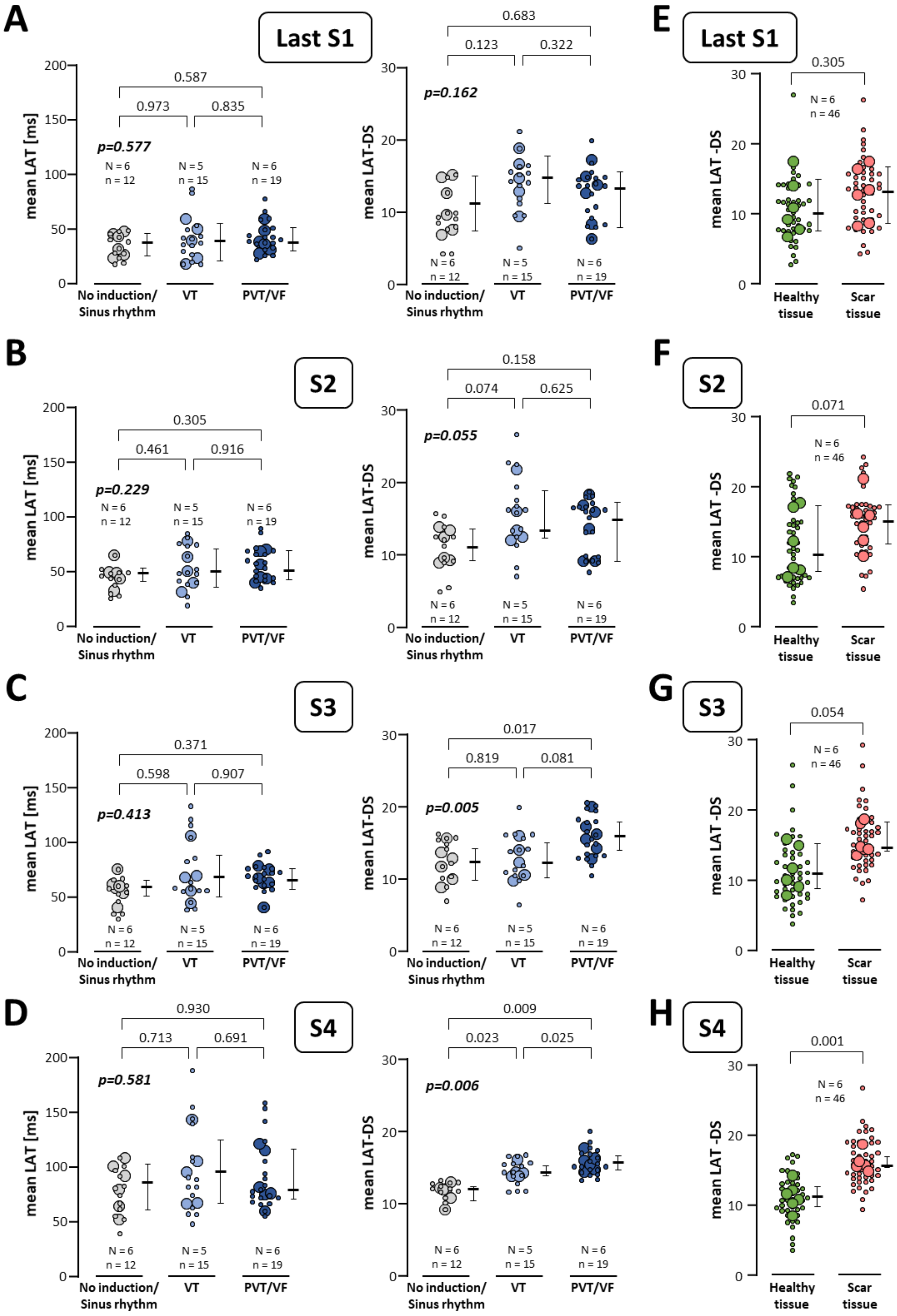

Figure S9. Average local activation time-dispersion score on single-beat maps and poststimulation arrhythmia severity. A-D, Quantification and comparisons of the average local activation times (LAT) and LAT-dispersion score (LAT-DS) during the last S1 **(A)**, S2 **(B)**, S3 **(C)** and S4 **(D)** coupled extrastimuli of the programmed ventricular stimulation (PVS) protocol based on the poststimulation outcome (i.e., no

induction/sinus rhythm, ventricular tachycardia [VT] and polymorphic VT [PVT]/ventricular fibrillation [VF]) in pigs from Group 2 (N=6). **E-H**, Quantification and comparisons of LAT-DS values between healthy and scar tissue at the time of the last S1 **(E)**, S2 **(F)**, S3 **(G)** and S4 **(H)** coupled extrastimuli of the PVS protocol. In all panels, each induction attempt is displayed with small colored circles ('n'), and the average of all the attempts for each animal and group are shown as large colored circles ('N'). Median and interquartile ranges alongside summarize the distribution of data displayed as large colored circles. Statistical analyses were performed using the data represented as large colored circles.
